# Toll-like receptor 8 agonist nanoparticles mimic immunomodulating effects of the live BCG vaccine and enhance neonatal innate and adaptive immune responses

**DOI:** 10.1016/j.jaci.2016.12.985

**Published:** 2017-11

**Authors:** David J. Dowling, Evan A. Scott, Annette Scheid, Ilana Bergelson, Sweta Joshi, Carlo Pietrasanta, Spencer Brightman, Guzman Sanchez-Schmitz, Simon D. Van Haren, Jana Ninković, Dina Kats, Cristiana Guiducci, Alexandre de Titta, Daniel K. Bonner, Sachiko Hirosue, Melody A. Swartz, Jeffrey A. Hubbell, Ofer Levy

**Affiliations:** a Department of Medicine, Division of Infectious Diseases, Boston Children's Hospital, Boston, Mass; b Harvard Medical School, Boston, Mass; c Department of Biomedical Engineering, Northwestern University, Evanston, Ill; d Division of Newborn Medicine, Floating Hospital for Children, Tufts Medical Center, Boston, Mass; e Neonatal Intensive Care Unit, Department of Clinical Sciences and Community Health, Fondazione IRCCS Ca' Granda Ospedale Maggiore Policlinico, University of Milan, Milan, Italy; f Dynavax Technologies, Berkeley, Calif; g Institute of Bioengineering, School of Life Sciences and School of Engineering, École Polytechnique Fédérale de Lausanne (EPFL), Lausanne, Switzerland; h Institute for Molecular Engineering, University of Chicago, Chicago, Ill; i *Precision Vaccine Program*, Division of Infectious Diseases, Boston Children's Hospital, Boston, Mass

**Keywords:** Newborn, dendritic cells, Toll-like receptor 8, polymersome, nanoparticle, BCG, vaccine, Ag85B, Antigen 85B, APC, Antigen-presenting cell, BMDC, Bone marrow–derived dendritic cell, DC, Dendritic cell, FITC, Fluorescein isothiocyanate, HBV, Hepatitis B vaccine, IMQ, Imidazoquinoline, LDH, Lactate dehydrogenase, MHCII, MHC class II, MoDC, Monocyte-derived dendritic cell, PCV, Pneumococcal conjugate vaccine, pDC, Plasmacytoid dendritic cell, PE, Phycoerythrin, PEG-*bl*-PPS, Poly(ethylene glycol)-bl-poly(propylene sulfide), PGE_2_, Prostaglandin E_2_, p25, Peptide 25, Tet^+^, Tetramer positive, TLR, Toll-like receptor, WT, Wild-type

## Abstract

**Background:**

Newborns display distinct immune responses, leaving them vulnerable to infections and impairing immunization. Targeting newborn dendritic cells (DCs), which integrate vaccine signals into adaptive immune responses, might enable development of age-specific vaccine formulations to overcome suboptimal immunization.

**Objective:**

Small-molecule imidazoquinoline Toll-like receptor (TLR) 8 agonists robustly activate newborn DCs but can result in reactogenicity when delivered in soluble form. We used rational engineering and age- and species-specific modeling to construct and characterize polymer nanocarriers encapsulating a TLR8 agonist, allowing direct intracellular release after selective uptake by DCs.

**Methods:**

Chemically similar but morphologically distinct nanocarriers comprised of amphiphilic block copolymers were engineered for targeted uptake by murine DCs *in vivo*, and a range of TLR8 agonist–encapsulating polymersome formulations were then synthesized. Novel 96-well *in vitro* assays using neonatal human monocyte-derived DCs and humanized TLR8 mouse bone marrow–derived DCs enabled benchmarking of the TLR8 agonist–encapsulating polymersome formulations against conventional adjuvants and licensed vaccines, including live attenuated BCG vaccine. Immunogenicity of the TLR8 agonist adjuvanted antigen 85B (Ag85B)/peptide 25–loaded BCG-mimicking nanoparticle formulation was evaluated *in vivo* by using humanized TLR8 neonatal mice.

**Results:**

Although alum-adjuvanted vaccines induced modest costimulatory molecule expression, limited T_H_-polarizing cytokine production, and significant cell death, BCG induced a robust adult-like maturation profile of neonatal DCs. Remarkably, TLR8 agonist polymersomes induced not only newborn DC maturation profiles similar to those induced by BCG but also stronger IL-12p70 production. On subcutaneous injection to neonatal mice, the TLR8 agonist–adjuvanted Ag85B peptide 25 formulation was comparable with BCG in inducing Ag85B-specific CD4^+^ T-cell numbers.

**Conclusion:**

TLR8 agonist–encapsulating polymersomes hold substantial potential for early-life immunization against intracellular pathogens. Overall, our study represents a novel approach for rational design of early-life vaccines.

## Graphical abstract

**Figure:**
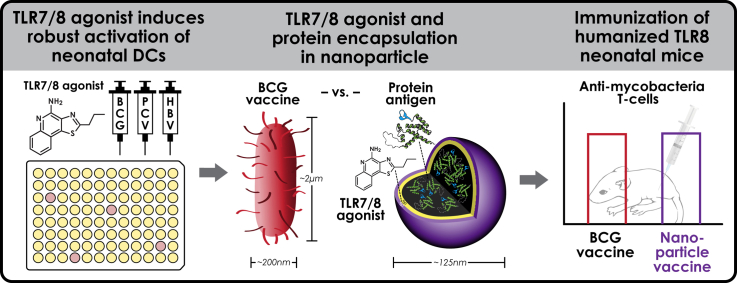

Human newborns and infants have a high frequency of infection compared with older children and adults,^1^ in part because of distinct early-life immunity with impaired host defense against intracellular pathogens.^2^ On challenge with many stimuli, including bacterial components, children less than 2 months of age express a strong innate T_H_2 and T_H_17 cell polarization and impaired T_H_1 cell and innate antiviral type 1 interferon responses.^3^ Relatively low innate T_H_1 cell support at birth appears to gradually increase over the first years of life.^2^ Distinct newborn T_H_17- and infant T_H_2-polarized responses potentially limit the efficacy of early-life immune response against certain pathogens^4^, ^5^ and vaccines.^6^, ^7^ Because birth is the most reliable point of health care contact worldwide, neonatal vaccines, such as the live attenuated BCG vaccine, achieve high global population penetration. Therefore developing early-life immunization strategies, including those directed at tuberculosis, might be highly advantageous.^8^

Dendritic cells (DCs) are professional antigen-presenting cells (APCs) that play a vital role in shaping adaptive immunity. DC maturation begins when endogenous or exogenous danger molecules are recognized by pattern recognition receptors (eg, Toll-like receptors [TLRs]), triggering upregulation of costimulatory molecules and production of immune-polarizing cytokines.^9^ Of note, human newborn DCs demonstrate impaired T_H_1 responses and particularly low production of TNF and IL-12p70, which are important for vaccine-induced protection against intracellular pathogens.^2^ Accordingly, development of novel adjuvanted vaccine formulations that enhance the maturation and functionality of human neonatal DCs might enable a new generation of early-life vaccines.^10^, ^11^ Unlike agonists of most TLRs that elicit reduced T_H_1 cytokine production by newborn leukocytes, agonists of the endosomal TLR8,^12^ such as the synthetic imidazoquinoline (IMQ) CL075 (TLR8/7 agonist), induce robust T_H_1-polarizing responses from both neonatal and adult DCs.^13^, ^14^

Recent advances in the nascent field of immunoengineering might guide vaccine design^15^, ^16^ by enabling targeting of DCs^17^ through vaccine delivery systems that mimic the size, shape, and surface chemistry of pathogens.^18^ Block copolymers of poly(ethylene glycol)-*bl*-poly(propylene sulfide) (PEG-*bl*-PPS) are new macromolecular amphiphiles capable of forming a wide range of self-assembled configurations when dispersed in water^19^ and might present distinct advantages for human vaccine development.^20^ PEG-*bl*-PPS aggregates can form a wide variety of stable morphologies in aqueous solution, including spherical or cylindrical micelles (filomicelles), as well as liposome-like vesicles referred to as polymersomes.^16^ Polymersomes are significantly more stable than liposomes and can be engineered for bioresponsive intracellular payload delivery,^16^ which is highly advantageous for the specific targeting of endosomal receptors. Polymersomes are effective adjuvant and antigen-delivery systems, particularly for the induction of T cell–mediated immunity and the encapsulation of IMQ-derived adjuvants.^21^, ^22^ Induction of durable T_H_1-type T-cell immunity might strengthen newborns' defenses, thereby reducing the morbidity and mortality associated with intracellular pathogens, such as respiratory syncytial virus, HIV, tuberculosis, and malaria.^23^, ^24^

We undertook an immunoengineering-based^16^ rational vaccine design^25^ approach to develop and characterize a novel polymersome nanocarrier encapsulating CL075, which was designated CL075-PS. CL075 has low water solubility and is a highly potent immunostimulant that can be systemically toxic. Accordingly, a targeted delivery system is likely beneficial for minimization of systemic toxicity and translational application. The *in vitro* immunostimulatory activities of CL075-PS on human newborn and adult monocyte-derived dendritic cells (MoDCs) were benchmarked against conventional adjuvants and human vaccines, including the live attenuated BCG vaccine, which elicits moderate T_H_1 immunity in neonates^26^ and is safe and effective at birth.^27^

Here we characterize the BCG-induced human DC cytokine and costimulatory molecule expression compared with conventional alum-adjuvanted vaccines and demonstrate that CL075-PS matches or exceeds this BCG-associated DC response.^28^ Compared with BCG, CL075-PS induced greater production of IL-12p70, a cytokine that enhances T_H_1-polarized immune responses and promotes cytotoxic T-cell proliferation and survival.^29^ The additional loading of the model HIV-1-Gag protein into CL075-PS did not negatively influence this response, triggering a similar protection-associated pattern of immune responses as induced by the whole bacterial vaccine BCG in newborn human DCs and greatly exceeding BCG-induced production of IL-12p70. When coloaded with the *Mycobacterium tuberculosis* antigen 85B (Ag85B) peptide 25 (p25), CL075:Ag85Bp25-PS induces Ag85B-specific CD4^+^ T cells in humanized TLR8 neonatal mice *in vivo*.

Our observations suggest a strong potential for CL075-PS to serve as a dual antigen/adjuvant vaccine delivery system for human neonatal vaccines. Moreover, our age- and species-specific approach for formulation development through benchmarking new adjuvant formulations with respect to DC activation and potential reactogenicity profiles of licensed vaccine formulations^30^ presents a new *in vitro* methodology for the rational design and testing of human newborn vaccines.

## Methods

### Ethics statement

All experiments were conducted in accordance with relevant institutional and national guidelines, regulations, and approvals. Nonidentifiable cord blood samples were collected with approval from the Ethics Committee of the Brigham & Women's Hospital, Boston, Massachusetts (protocol no. 2000-P-000117), and Beth Israel Deaconess Medical Center Boston, Boston, Massachusetts (protocol no. 2011P-000118). Blood samples from adult volunteers were obtained after written informed consent with approval from the Ethics Committee of Boston Children's Hospital, Boston, Massachusetts (protocol no. X07-05-0223). All murine studies were approved by the Institutional Animal Care and Use Committee at Harvard Medical School and Boston Children's Hospital (protocol nos. 3011 and 3130).

### *In vivo* nanocarrier biodistribution

PEG-*bl*-PPS was amine functionalized at the end of the polypropylene sulfide block for conjugation to the fluorophore Dy647-N-hydroxysuccinimidyl ester (Dyomics GmbH, Jena, Germany). After purification with Sephadex LH-20 resin and dialysis, the fluorescent block copolymer was self-assembled into micelles, polymersomes, or filomicelles through thin-film rehydration. C57BL/6 mice were injected intradermally in the footpad with 30 μL of a 5 mg/mL solution of the fluorescent nanocarriers in PBS (120 μL in total volume). After 24 hours, the mice were killed, and the popliteal, inguinal, axillary, and brachial lymph nodes along with the spleen were harvested and digested with collagenase D.

Preparations of single-cell suspensions were obtained after filtration of digested organs through a 70-μm strainer (Miltenyi Biotec, Auburn, Calif). Cells were stained with the following antibody panels: (1) CD207–fluorescein isothiocyanate (FITC), MHC class II (MHCII)–phycoerythrin (PE), CD11b-Cy5.5, CD11c-Cy7, CD103–Pacific Blue, and CD8α–allophycocyanin Cy7 and (2) MHCII-FITC, NK1.1-PE, CD19-PE, Ly6c-Cy5.5, CD11b-Cy7, and CD11c–allophycocyanin Cy7. Flow cytometry was performed with a CyAn ADP analyzer (Beckman Coulter, Nyon, Switzerland).

### Human MoDC arrays

Heparinized human newborn cord blood or adult peripheral blood was layered onto Ficoll-Hypaque gradients (Ficoll-Paque PREMIUM; GE Healthcare, Waukesha, Wis) to collect cord blood mononuclear cell or PBMC layers, respectively. Monocytes were isolated from mononuclear cell fractions by means of positive selection with magnetic microbeads, according to the manufacturer's instructions (Miltenyi Biotec), with CD14 as a panmarker. Monocyte preparations were routinely more than 95% pure, as assessed by using flow cytometry for CD14, as previously described.^13^, ^14^, ^31^, ^32^ Isolated monocytes were cultured in tissue-culture dishes at 0.4 × 10^6^ cells/mL in RPMI 1640 medium containing fresh 10% autologous platelet-poor plasma supplemented with recombinant human IL-4 (50 ng/mL) and recombinant human GM-CSF (100 ng/mL; R&D Systems, Minneapolis, Minn) with 1 supplement of fresh medium and cytokines at day 3 of culture. After 5 to 6 days, immature MoDCs, routinely HLA-DR^+^CD14^−^ DC-specific intercellular adhesion molecule 3–grabbing nonintegrin (DC-SIGN)^+^,^13^ of greater than 90% purity were harvested by gently pipetting only the loosely adherent fraction and replated in a 96-well format at the desired cell density. Human neonatal and infant vaccines and adjuvants used for benchmarking included BCG Vaccine Danish Strain 1331 (Statens Serum Institut, Copenhagen, Denmark), RECOMBIVAX HB Hepatitis B Vaccine (HBV; Merck & Co, Whitehouse Station, NJ), Pneumococcal 13-valent Conjugate Vaccine ([Diphtheria CRM197 Protein]; Pfizer, New York, NY) and AdjuPhos AlPO_4_ (Brenntag Biosector, Frederikssund, Denmark).

### Cell viability, ELISA, and multiplex analyte assays

Supernatants derived from human DC stimulations were assayed by means of ELISA for TNF (BD Biosciences, San Jose, Calif). Cytokine and chemokine expression profiles (for IFNγ, IL-9, IL-10, IL-12 [p70], IL-13, IL-1β, IL-23, IL-27, IL-28A, IL-33, IL-6, macrophage inflammatory protein 3α/CCL20, and TNF) in cell-culture supernatants were measured with a customized Milliplex Human Th17 Magnetic Bead Panel, according to the manufacturer's instructions (Millipore, Chicago, Ill). Assays were read and analyzed on the Luminex 100/200 System with xPOTENT software (Luminex, Austin, Tex). A minimum threshold was set at the minimum detectable concentration for each individual assay (defined as 3 SDs greater than the mean background). Cell death of treated MoDCs was assessed based on release of active lactate dehydrogenase (LDH) into cell supernatants with a Cytotoxicity Detection Kit, according to the manufacturer's instructions (Roche Diagnostics, Branford, Conn). Prostaglandin E_2_ (PGE_2_) concentrations in culture supernatants were determined by using a monoclonal enzyme immunoassay (Cayman Chemical Company, Ann Arbor, Mich), with the analytic tool provided by Cayman Chemical Company at www.myassays.com.

### Immunizations

C57BL/6 wild-type (WT) dams and heterozygous humanized TLR8 (huTLR8Tg) male mice on a C57BL/6 background^33^ were bred to produce mixed litters. All littermates from one litter were immunized with the same formulations to eliminate the possibility of misidentification. Mice were immunized on day 7 of life and killed 14 days later. Genotyping was performed with tail clippings at 21 days of life. Mice were immunized subcutaneously at the scruff of the neck with either 0.2 × 10^6^ colony-forming units of BCG (Organon Teknika/Merck, Durham, NC) or CL075:Ag85Bp25-PS containing approximately 8 to 10 μg of Ag85B/p25 (Biomatik, Cambridge, Ontario, Canada) and approximately 0.1 mg/kg CL075 in a total volume of 25 μL. For flow cytometric analysis, spleens were collected, and a single-cell suspension was generated and blocked with FcR blocking anti-mouse CD16/CD32 mAb 2.4G2 at 4°C for 10 minutes and then stained for 1 hour at room temperature with PE-conjugated I-A(b) *M tuberculosis* Ag85B precursor 280-294 (FQDAYNAAGGHNAVF) or human class II–associated invariant chain peptide (PVSKMRMARPLLMQA) tetramers (kindly produced by the National Institutes of Health MHC Tetramer Core Facility, Emory University, Atlanta, Ga), as previously described.^34^ Surface staining was performed at 4°C for 30 minutes with FITC-conjugated anti-CD3 (clone 17A2), allophycocyanin-conjugated anti-CD4 (clone RMA-5), BV421-conjugated anti-CD44 (clone IM7), and PE-conjugated anti-CD62L (clone SB/199, all from BD Biosciences). Samples were also labeled with Live/Dead Fixable Near IR Dead Cell Stain Kit, according to the manufacturer's instructions (Invitrogen, Carlsbad, Calif). CD44^+^ tetramer-positive (Tet^+^) cells were identified after gating for singlets, forward scatter × side scatter lymphocyte characteristics, live cells, and CD4 expression. About 10^6^ cells were stored for each sample acquired on the LSR II flow cytometer by using BD FACSDiva software (BD Biosciences, San Jose, Calif); data analysis was performed with FlowJo software (TreeStar, Ashland, Ore).

### Statistical analysis

Statistical significance and graphic output were generated with Prism (version 5.0b; GraphPad Software) and Microsoft Excel (Microsoft, Redmond, Wash) software. Statistical tests are stated in figure legends. Results were considered significant at *P* values of less than .05.

## Results

### Selection of nanocarrier morphology based on *in vivo* biodistribution

We have previously demonstrated that PEG-*bl*-PPS block copolymers can self-assemble in aqueous solutions into a variety of different morphologies by adjusting the ratios of the sizes of the hydrophilic (PEG) and hydrophobic (PPS) blocks.^19^, ^20^ Copolymers with hydrophilic block weight fractions of 0.28, 0.38, and 0.48 respectively, assemble into vesicles also known as polymersomes, filomicelles, and spherical micelles that mimic the diverse nanostructures of virions.^21^, ^22^ We hypothesized that the use of pathogen-mimicking nanostructures might enhance *in vivo* targeting and uptake by DCs, which have evolved to endocytose and process nanoscale materials from infective agents for the elicitation and direction of immune responses.

**Fig 1:**
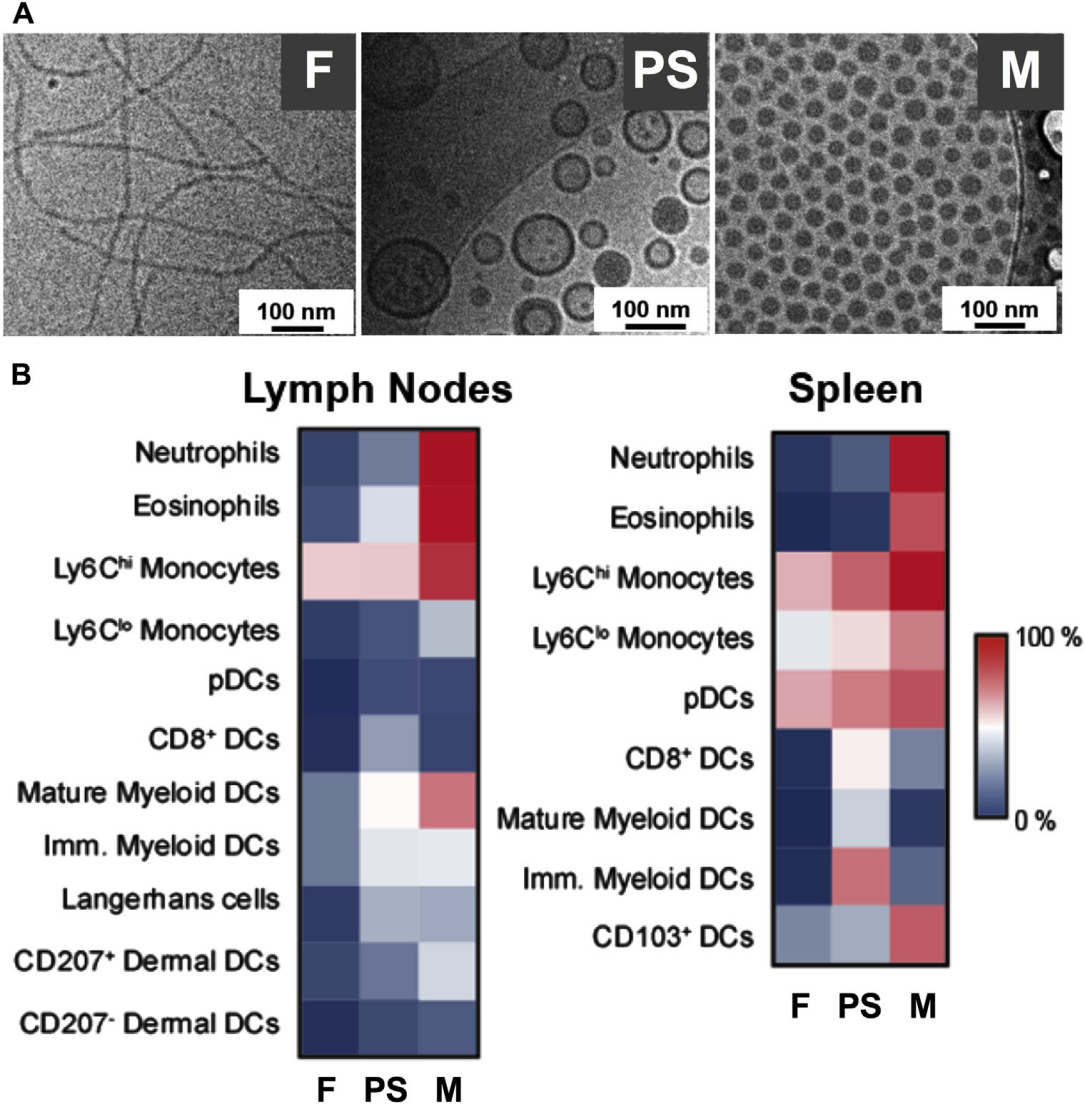
Polymersomes preferentially associate with DCs *in vivo*. **A,** Cryogenic transmission electron microscopy revealed the self-assembly of approximately 50-nm filomicelles (*F*; *left*, PEG_45_-*bl*-PPS_44_), approximately 120-nm vesicular polymersomes (*PS*; *middle*, PEG_17_-*bl*-PPS_30_), or approximately 30-nm spherical micelles (*M*; *right*, PEG_44_-*bl*-PPS_29_). **B,** Flow cytometric analysis of single-cell suspensions from mouse lymph nodes and spleens was conducted 24 hours after subcutaneous injection of fluorescent nanocarriers into the footpad (n = 5-8). A heat map demonstrates percentages of key phagocyte populations that associated with PEG-*bl*-PPS filomicelles *(F)*, vesicular polymersomes *(PS)*, and spherical micelles *(M)*. Amine-functionalized PEG_17_-*bl*-PPS_30_ block copolymers were conjugated to fluorescent Dy647-N-hydroxysuccinimide and assembled in the presence of block copolymers engineered to generate filamentous, vesicular, or spherical morphologies. See also Fig E1.

To identify the optimal morphology for *in vivo* targeting of diverse DC populations while minimizing nonspecific cellular uptake, we compared the biodistributions of these PEG-*bl*-PPS morphologies within the spleen and injection site–draining lymph nodes, which are relevant DC-rich secondary lymphoid organs essential to vaccination. Amine-functionalized PEG_17_-*bl*-PPS_30_ block copolymers were conjugated to fluorescent Dy647-N-hydroxysuccinimide and assembled in the presence of block copolymers engineered to form polymersomes (PEG_17_-*bl*-PPS_30_), filomicelles (PEG_45_-*bl*-PPS_44_), or spherical micelles (PEG_44_-*bl*-PPS_29_). Cryogenic transmission electron microscopy revealed the self-assembly of fluorescent 120-nm polymersomes, 50-nm diameter by micron length filomicelles, and 30-nm spherical micelles (Fig 1).

**Fig E1:**
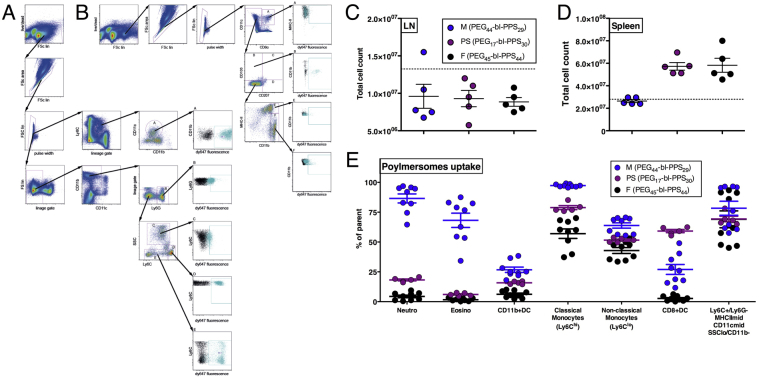


Dy647-labeled aggregates were injected subcutaneously into the footpads of mice, and 24 hours later, leukocytes were isolated from spleens and lymph nodes for analysis by using flow cytometry. Noticeably, even though the surface chemistry of the different PEG-*bl*-PPS aggregates is identical, different morphologies associated with drastically different monocytic cell subsets (Fig 1, *B*, and see Fig E1 in this article's Online Repository at www.jacionline.org). These results strongly indicate the influence of physical structure and size of the various aggregates on their cellular biodistribution, suggesting that rate of transport, accessibility of the aggregates to different organs, and cell-specific mechanisms of endocytosis affect their targeting of immune cell subsets. Although the micelles were extensively taken up by numerous phagocytic subpopulations in lymph nodes and spleens, including potentially undesirable high percentages of neutrophils and eosinophils, the filomicelles were found to primarily target monocytes in the lymph nodes and spleen and plasmacytoid dendritic cells (pDCs) in the spleen (Fig 1, *B*). In the spleen more than 50% of the Ly6C^hi^ or Ly6C^lo^ monocyte population and up to 95% of the pDC population were fluorescent for the filomicelles. In addition to pDCs, the polymersome nanocarriers targeted numerous other DC subpopulations, including approximately 50% of the splenic and approximately 30% of the lymph node–resident CD8^+^ DCs. Uptake of all aggregates was restricted to CD45^+^ cells, suggesting minimal to no uptake by stromal cells. Based on these results, we selected the polymersome nanomaterial morphology for targeting of CL075 to key DC populations while minimizing nonspecific uptake after subcutaneous vaccine administration.

### Synthesis, loading, and characterization of CL075-PS

**Fig 2:**
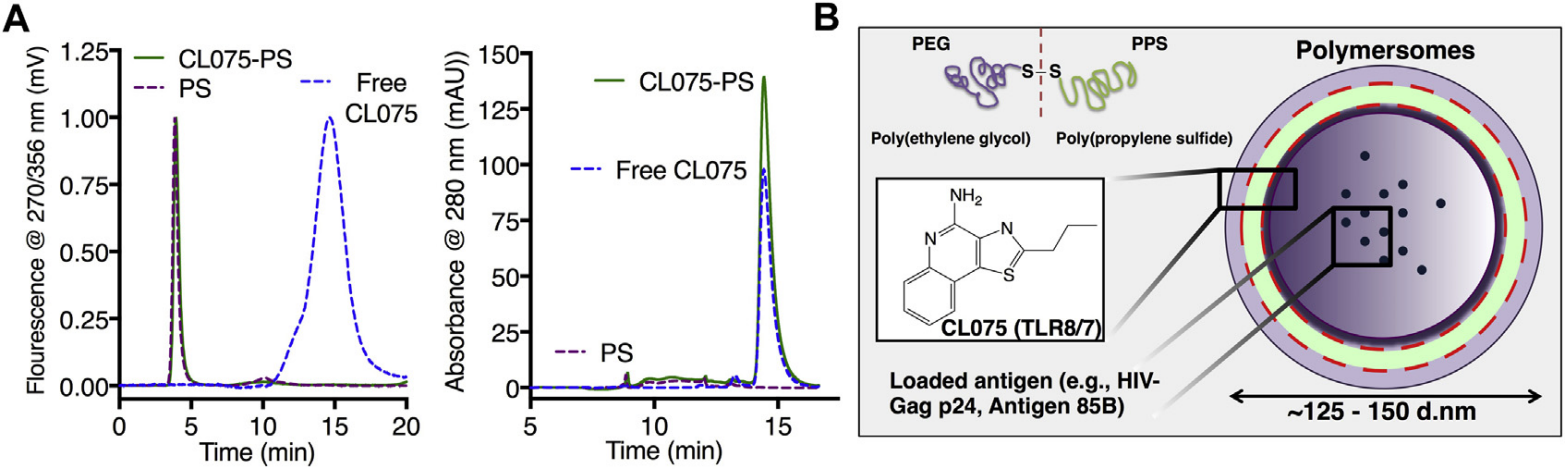
Synthesis, loading, and characterization of CL075-PS. **A,***Left*, Size exclusion chromatography with Sepharose CL-6B resin and a PBS mobile phase indicated there was no free CL075 present after purification of the CL075-loaded polymersomes. The *green line* indicates a strong peak for the polymersomes, which elute very early, without a second peak for CL075 *(dashed blue line)*. Data are plotted against blank unloaded polymersomes, demonstrating the location of the polymer *(purple line)*. *Right*, CL075 dissolved readily in the solvent dimethylformamide, as did the PEG-PPS block copolymer used to assemble polymersomes. CL075-PS was lyophilized so that both the polymer and adjuvant were completely dissolved in the dimethylformamide. Both free *(dashed blue line)* and loaded CL075 *(green line)* eluted at approximately 14.48 minutes. **B,** Graphic depiction of the CL075-PS nanoparticles. See also Figs E2-E4.

**Fig E2:**
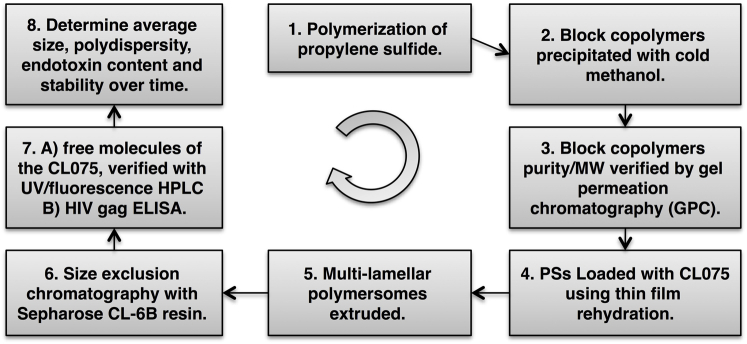


**Fig E3:**
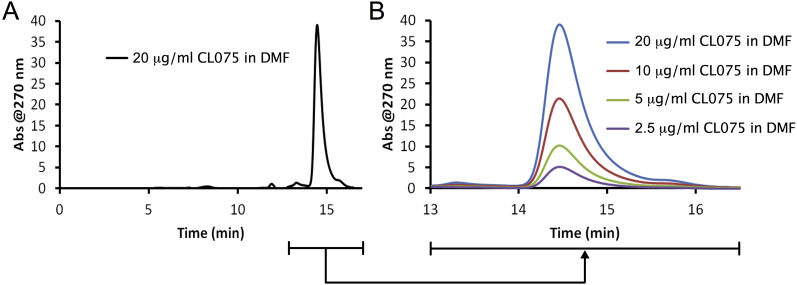


A range of polymersome formulations was synthesized to assess their efficacy for DC activation, including blank (unloaded) polymersomes and polymersomes loaded with CL075, which we designate CL075-PS, the capsid protein antigen HIV-1-Gag, Ag85Bp25, and both CL075 and protein/peptide simultaneously (Table I). CL075-PS nanoparticles were assembled by using thin-film rehydration and purified by means of size exclusion chromatography (outlined in Fig E2 in this article's Online Repository at www.jacionline.org). Optimal loading of CL075 into polymersomes was observed with encapsulation efficiencies of greater than 90% when codissolving CL075 and PEG_17_-*bl*-PPS_30_ in a mutual organic solvent before desiccation and formation of the thin-film. Analysis of size exclusion chromatography elutions by means of UV/fluorescence spectroscopy with a PBS mobile phase verified that no free CL075 was present after polymersome purification (Fig 2, *A*). Encapsulated CL075 was quantified by means of UV/fluorescence HPLC against known standards with a dimethylformamide mobile phase (see Fig E3 in this article's Online Repository at www.jacionline.org). CL075-PS formulations (as illustrated in Fig 2, *B*) with concentrations of up to 5.4 mmol/L CL075 were achieved with loading efficiencies of 10 μg of CL075/1 mg of PEG-*bl*-PPS (CL075:PEG-*bl*-PPS molar ratio of 1:9).

**Table I:** Polymersome details, loading, and purification

Name	Loaded IMQ	TLR	Maximum loaded IMQ concentration (mmol/L)	PEG-PPS concentration (mg/mL)	Average diameter (nm)	PdI index	Endotoxin-LAL assay (EU/mL)	Endotoxin-TLR4 assay (EU/mL)
Polymersomes	NA	NA	NA	32.9	156	0.111	<1	<0.030
CL075-PS	CL075	8	1.4	34.2	120	0.171	<1	<0.030
CL075:Gag-PS	CL075	8	1.4	33.2	155	0.140	<1	<0.030
CL075:Ag85Bp25-PS	CL075	8	5.4	20.8	148	0.108	<1	<0.030

*NA*, Not applicable; *PdI*, polydispersity; *PEG*, polyethylene glycol; *PEG-PPS*, poly(ethylene glycol)-bl-poly(propylene sulfide); *PPS*, polypropylene sulfide; *PS*, polymersome.

**Fig E4:**
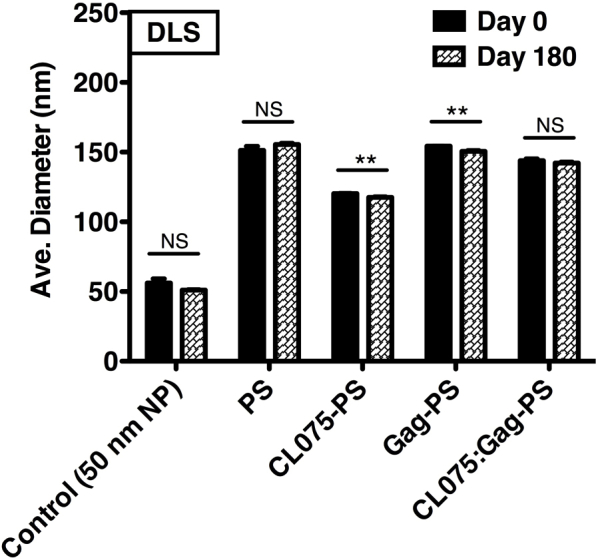


Loading was found to be highly stable, with no leakage of CL075 detectable (detection limit, approximately 1 ng/mL) for more than 1 year at 4°C in PBS (see Fig E2 and data not shown). HIV-1-Gag protein was stably loaded into polymersomes by using previously published protocols for protein antigens.^21^ Each batch of polymersome was characterized with respect to quantification of the amount of loaded protein/peptide, PEG-*bl*-PPS concentration, average size, polydispersity, and endotoxin content (Table I). Additionally, when stored in PBS at 4°C, polymersome formulations were routinely stable in size for more than 180 days (see Fig E4 in this article's Online Repository at www.jacionline.org), with only CL075-PS and Gag-PS demonstrating minor size changes (approximately 120 to approximately 117 and approximately 154 to approximately 151 nm in diameter, respectively; *P* < .01).

### CL075-PS matches or exceed BCG-induced activation of human newborn DCs

**Fig 3:**
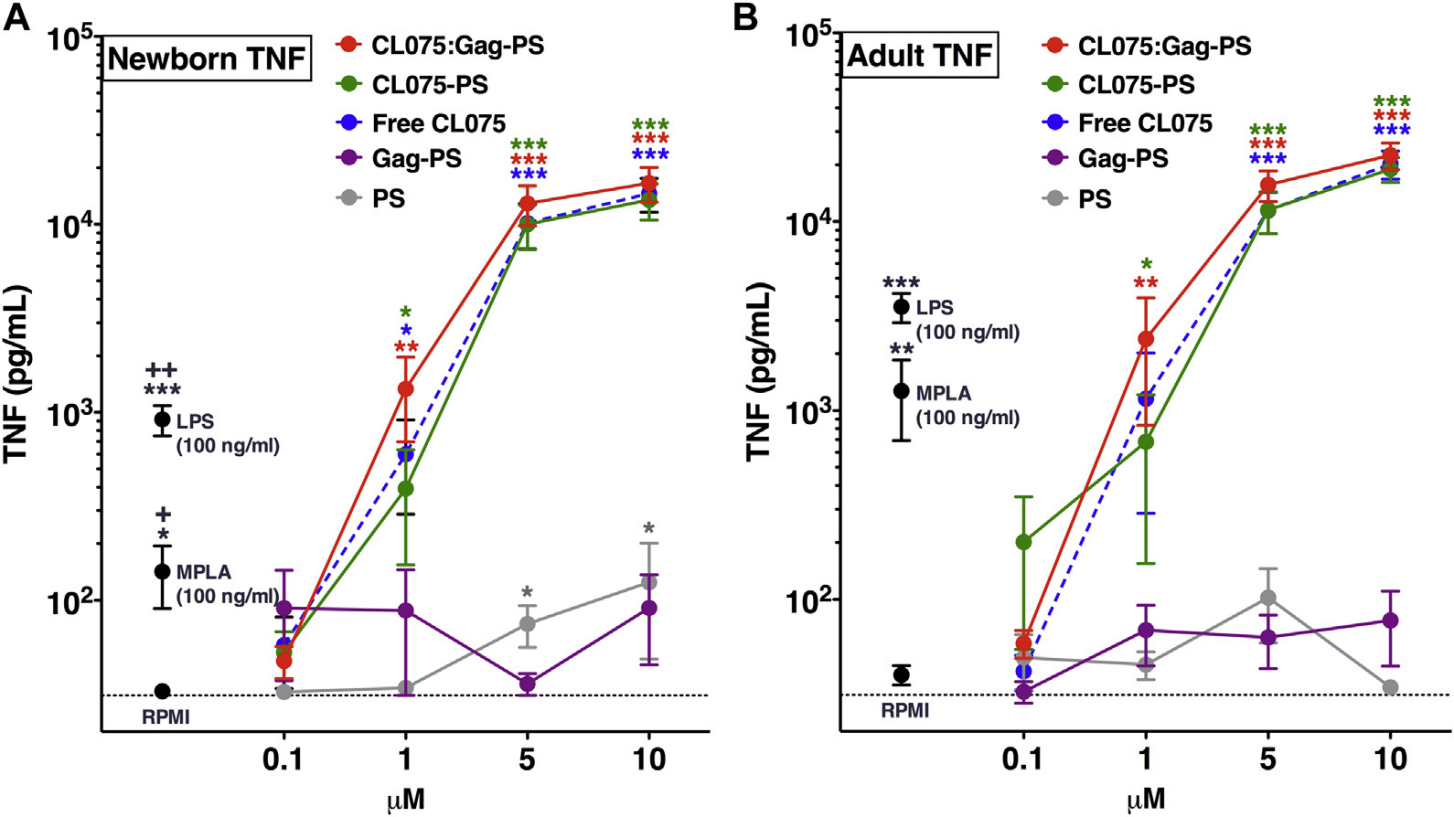
The TLR8 agonist CL075 retains robust TNF-inducing activity toward human newborn and adult MoDCs when encapsulated in polymersome nanoparticles. Human newborn **(A)** and adult **(B)** MoDCs were cultured in 10% (vol/vol) autologous plasma and stimulated for 24 hours with fixed concentrations of monophosphoryl lipid A *(MPLA)* or LPS (100 ng/mL) or increasing concentrations of free CL075 *(dashed blue line)*, CL075-PS *(green line)*, or CL075:Gag-PS (*red line*; mean ± SEM, n = 8-14). For analyses of individual treatments (eg, control RPMI vs 10 μmol/L CL075), the unpaired Mann-Whitney test was applied at each concentration, and statistical significance was denoted as follows: *P < .05, **P < .01, and ***P < .001. For comparisons between newborn and adult TLR4 agonist–treated conditions (ie, newborn LPS vs adult LPS), significance was denoted as follows: +*P* < .05 and ++*P* < .01. See also Fig E6.

**Fig E5:**
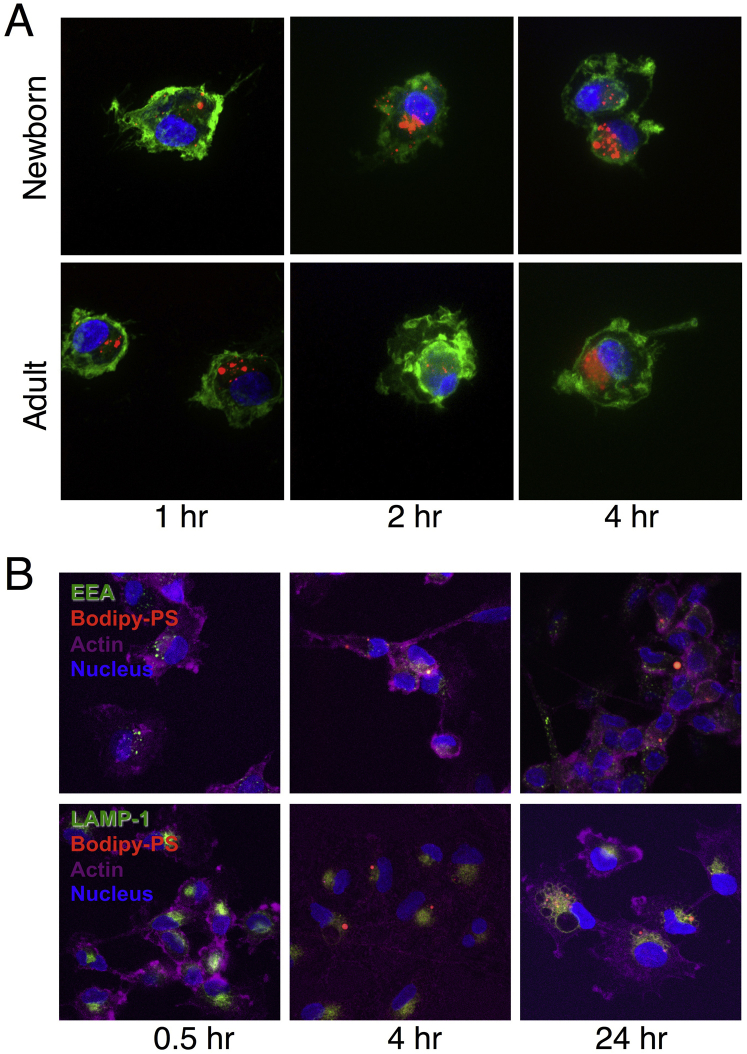


**Fig E6:**
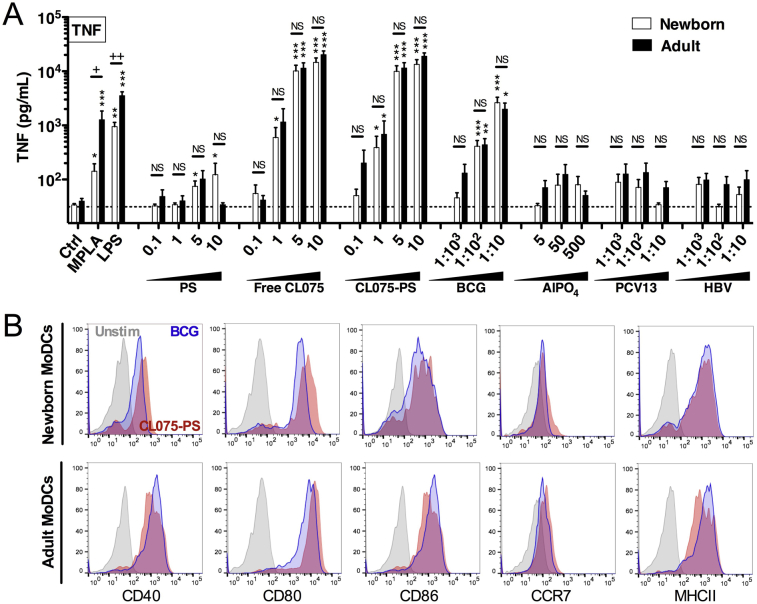


Ninety-six-well human MoDC arrays were generated by culturing CD14-selected monocytes with IL-4 and GM-CSF in the presence of autologous plasma, a rich source of age-specific soluble immunomodulatory factors,^35^ as previously described.^14^, ^31^ Within 30 minutes after addition to DCs, both newborn and adult immature DCs internalized fluorescent polymersomes in a concentration-dependent manner (see Fig E5, *A*, and Video E1 in this article's Online Repository at www.jacionline.org and data not show). Furthermore, polymersomes colocalized with late endosomal/lysosomal compartments within 24 hours (see Fig E5, *B*). Next, immature newborn and adult DCs were incubated with the adjuvant monophosphoryl lipid A, LPS, CL075, or CL075-PS for 24 hours (Fig 3). Newborn DCs demonstrated significantly reduced TLR4-mediated TNF responses (*P* < .05) but adult-like TNF production in response to the CL075-encapsulating polymersome formulation (Fig 3 and see Fig E6 in this article's Online Repository at www.jacionline.org). Interestingly, encapsulation of CL075 did not interfere with agonist activity, as evident by the concentration-dependent activity toward both newborn and adult DCs.

**Fig 4:**
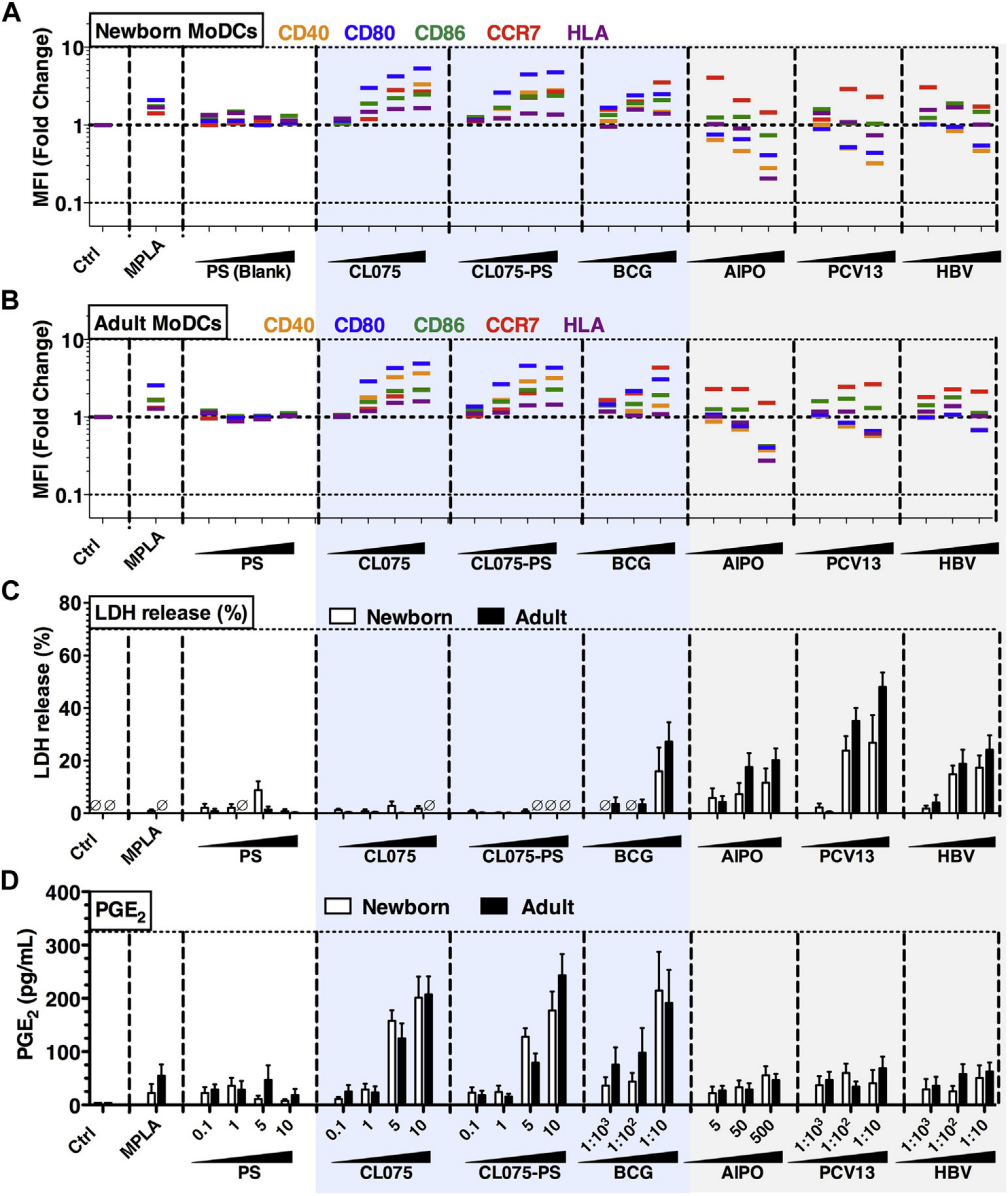
CL075-PS induces a robust MoDC activation profile similar to that of BCG and distinct from that of alum-adjuvanted subunit vaccines. **A** and **B,** Newborn (Fig 4, *A*) and adult (Fig 4, *B*) MoDCs were cultured in 10% (vol/vol) autologous plasma and stimulated for 24 hours with fixed concentrations of monophosphoryl lipid A (*MPLA*; 100 ng/mL), CL075, CL075-PS (both 0.1, 1, 5, and 10 μmol/L), alum (5, 50, and 500 μg/mL), BCG, PCV13, or HBV (each at 1:1000, 1:100, and 1:10 vol/vol). Surface expression of costimulatory molecules and HLA was determined by using flow cytometry and analyzed as fold change in mean fluorescent intensity *(MFI)* versus vehicle control (mean ± SEM, n = 5-8). **C,** Neither polymersomes *(PS)*, CL075, nor CL075-PS induced cell death. Undetectable LDH levels are indicated by a *circle with a diagonal line through it*. **D,** PGE_2_ production, as measured by means of ELISA, benchmarked to adjuvants and licensed pediatric vaccines (mean ± SEM, n = 6). See also Figs E7 and E8 and Table E1.

Our unique 96-well MoDC array platform next enabled comparison, for the first time, of concentration-dependent human newborn and adult MoDC responses to BCG and alum, as well as conventional alum-adjuvanted vaccines, such as HBV and pneumococcal conjugate vaccine (PCV; Fig 4, *A* and *B*, and see Table E1 in this article's Online Repository at www.jacionline.org). These induced distinct costimulatory molecule expression, with BCG inducing greater upregulation of MHCII, CD40, CD80, and CD86 than observed in the alum group. When benchmarked against conventional adjuvants and licensed pediatric vaccines in the MoDC array, CL075-PS induced greater TNF production than the alum-containing vaccines (HBV and PCV13), which was very similar to BCG (see Fig E6, *A*). The ability of TLR8 agonist–encapsulating polymersomes to mimic the immunomodulatory effect of the live BCG vaccine toward neonatal DCs extended to the surface expression of costimulatory molecules (Fig 4, *A* and *B*, and see Fig E6, *B*, and Table E1). In response to CL075, CL075-PS, or BCG, a strong upregulation of CD40, CD86, and CCR7 expression and a modest increase in MHCII expression was observed. The upregulation of CD80 induced by both CL075-containing formulations was greater than that induced by BCG. CL075-PS did not induce as much LDH release as did BCG, suggesting lower potential for cell death/cytotoxicity (Fig 4, *C*), whereas PGE_2_ production (Fig 4, *D*), a potential biomarker for reactogenicity,^36^ was similar that seen with the well-tolerated BCG.

**Fig 5:**
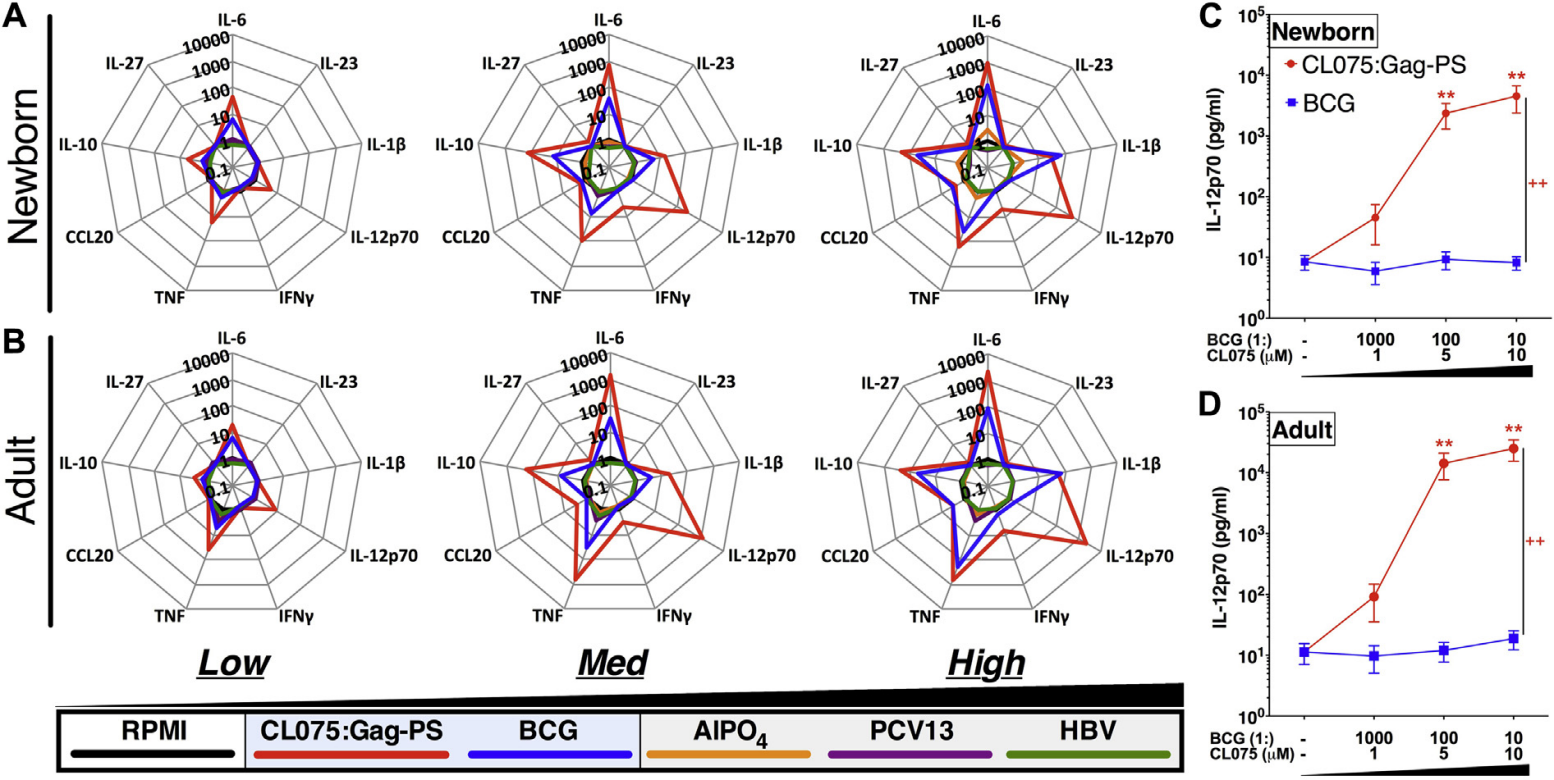
TLR8 agonist–encapsulating polymersomes match or exceed BCG's cytokine-inducing activity, dramatically enhancing human neonatal and adult MoDC IL-12p70 production. Newborn (**A** and **C**) and adult (**B** and **D**) MoDCs were cultured in 10% (vol/vol) autologous plasma and stimulated for 24 hours with increasing concentrations of CL075:Gag-PS (*red*; 1, 5, and 10 μmol/L), BCG *(blue)*, PCV13 *(purple)*, HBV (*green*; each at 1:1000, 1:100, and 1:10 vol/vol), or alum (5, 50, 500 and μg/mL; *orange*). Concentration-dependent cytokine production induction was measured in supernatants by using the multiplex assay. Data represent fold change over vehicle control (*black*; mean ± SEM, n = 6). For analyses of individual treatments (eg, control 10 μmol/L CL075:Gag-PS vs BCG 1:10 [vol/vol]), the unpaired Mann-Whitney test was applied at each concentration, and statistical significance was denoted as follows: **P < .01. For comparisons between overall groups (eg, CL075:Gag-PS vs BCG), significance was denoted as follows: ++*P* < .01. See also Figs E9 and E10.

**Fig E7:**
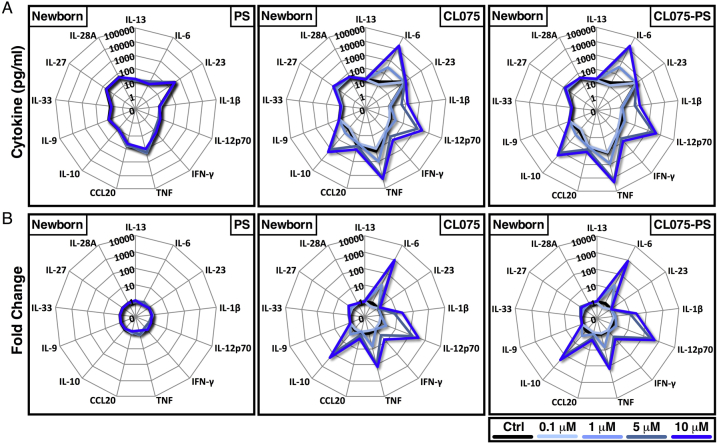


**Fig E8:**
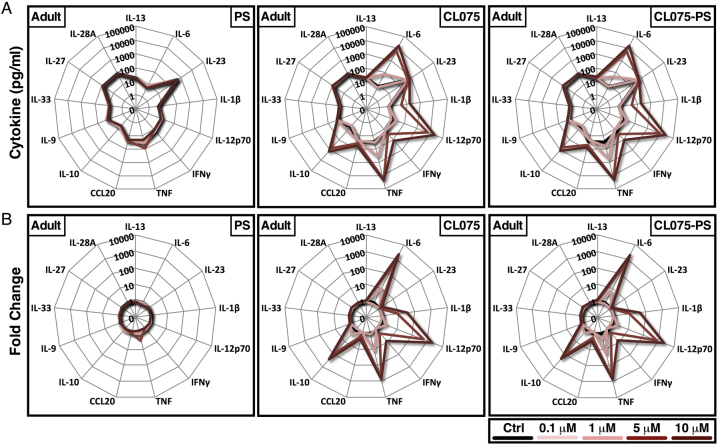


**Fig E9:**
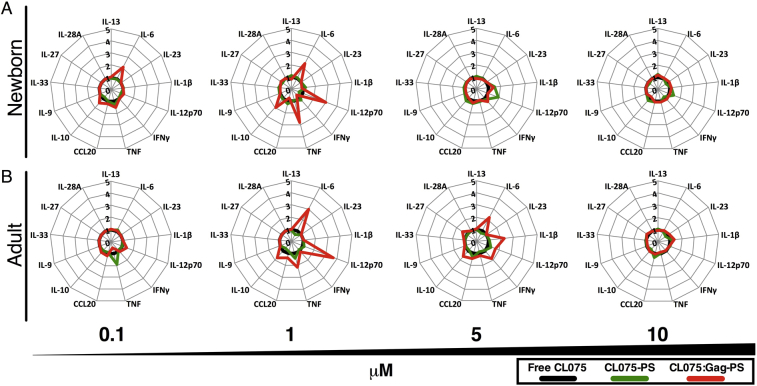


**Fig E10:**
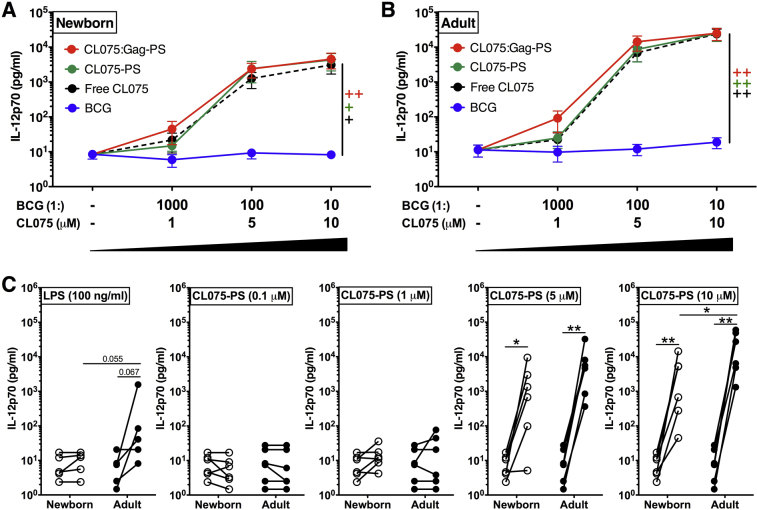


We next characterized the ability of polymersomes, CL075, or CL075-PS (the latter 2 at 0.1-10 μmol/L CL075) to induce concentration-dependent cytokine production from newborn and adult DCs (see Figs E7 and E8 in this article's Online Repository at www.jacionline.org). As expected, polymersomes alone did not induce significant cytokine production for any T_H_ cell–polarizing cytokines, whereas CL075 and CL075-PS induced concentration-dependent production of IL-1β, IL-6, IL-10, IL-12p70, and TNF. When compared head to head, CL075, CL075-PS, and CL075:Gag-PS generated markedly similar cytokine production profiles (see Figs E9 and E10, *A* and *B*, in this article's Online Repository at www.jacionline.org). Moreover, when benchmarked against conventional adjuvants and vaccines, both newborn and adult DCs treated with CL075:Gag-PS induced cytokine responses strikingly resembling the polarization and magnitude of those induced by BCG alone (Fig 5, *A* and *B*). Alum and the alum-adjuvanted HBV and PCV induced limited or no cytokine production. However, in comparing CL075:Gag-PS with BCG, 2 differences were noted: (1) CL075:Gag-PS induced greater production of IL-12p70 (at both 5 and 10 μmol/L, *P* < .01) in both newborn and adult DCs (Fig 5, *C* and *D*, and see Fig E10, *A* and *B*), and (2) a similar pattern was observed for IFNγ at the highest concentrations tested (*P* < .01, Fig 5, *A*; *P* < .05, Fig 5, *B*). Additionally, although LPS-induced IL-12p70 production was higher in adult DCs, significant differences between CL075-PS–stimulated MoDCs from adults and newborns were only evident at the 10 μmol/L concentration (see Fig E10, *C*).

### CL075:Ag85Bp25-PS induce Ag85B-specific CD4^+^ T cells in humanized TLR8 neonatal mice

**Fig E11:**
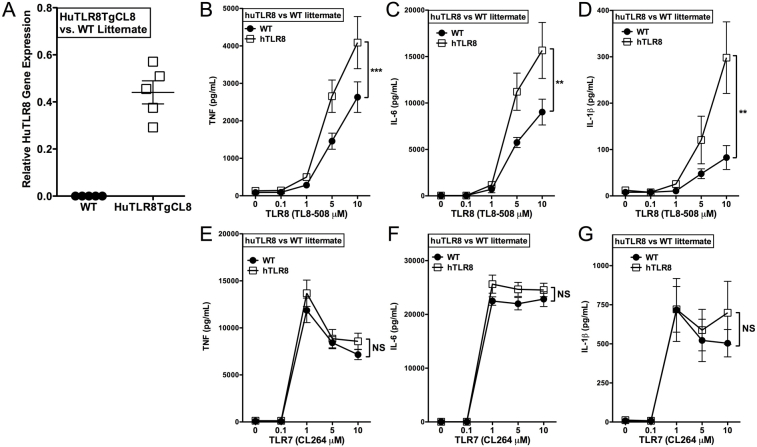


**Fig E12:**
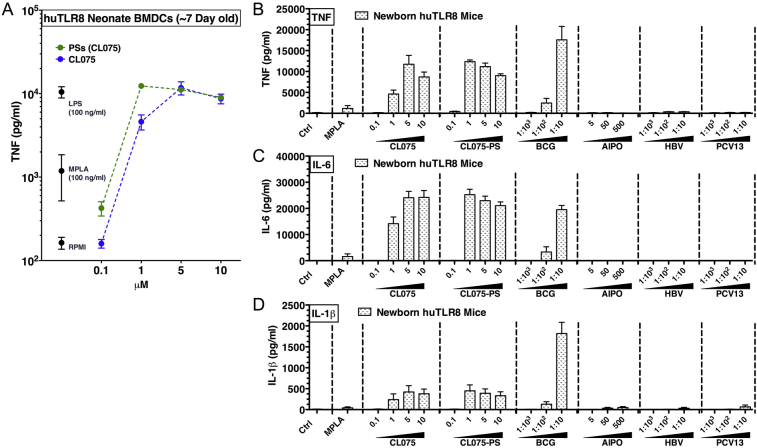


Having demonstrated nanocarrier morphology-dependent *in vivo* biodistribution, as well as CL075-PS–induced activation of human DCs *in vitro*, we next aimed to evaluate the potential of antigen-loaded CL075-PS formulations *in vivo*. Of note, evaluation of TLR8 agonist–adjuvanted vaccine formulations in mice is complicated by the fact that murine TLR8 does not recognize the same agonists as human TLR8.^37^ To account for this species specificity of TLR8 function, we assessed the effect of antigen-loaded CL075-PS on the *in vitro* activation of murine DCs, as well as induction of antigen-specific immunogenicity using neonatal humanized TLR8 (huTLR8Tg) mice.^33^ huTLR8Tg mice are an excellent model to evaluate our CL075-PS formulations because both the function and cellular distribution of human TLR8 in these transgenic mice is similar to that in human subjects.^33^ When compared with bone marrow–derived dendritic cells (BMDCs) of WT littermates, BMDCs from 7-day-old neonatal huTLR8Tg mice, which selectively express the gene for human TLR8 (see Fig E11, *A*, in this article's Online Repository at www.jacionline.org), had a significantly greater innate immune response to TLR8-selective stimulation (see Fig E11, *B-D*), whereas the response to a TLR7 agonist was similar (see Fig E11, *E-G*). To directly assess whether CL075-PS–stimulated murine BMDC responses align with our results observed in human MoDCs, we stimulated 7-day-old newborn-derived BMDCs for 24 hours with fixed concentrations of monophosphoryl lipid A (100 ng/mL), free CL075, CL075-PS (see Fig E12, *A*, in this article's Online Repository at www.jacionline.org), BCG, or alum-adjuvanted vaccines before collection of supernatants for TNF, IL-6, and IL-1β measurement (see Fig E12, *B-D*). These murine BMDC data recapitulate the results from our human MoDC experiments, suggesting that newborn huTLR8Tg mice represent a rational model for assessing the potential of CL075-PS *in vivo*.

**Fig 6:**
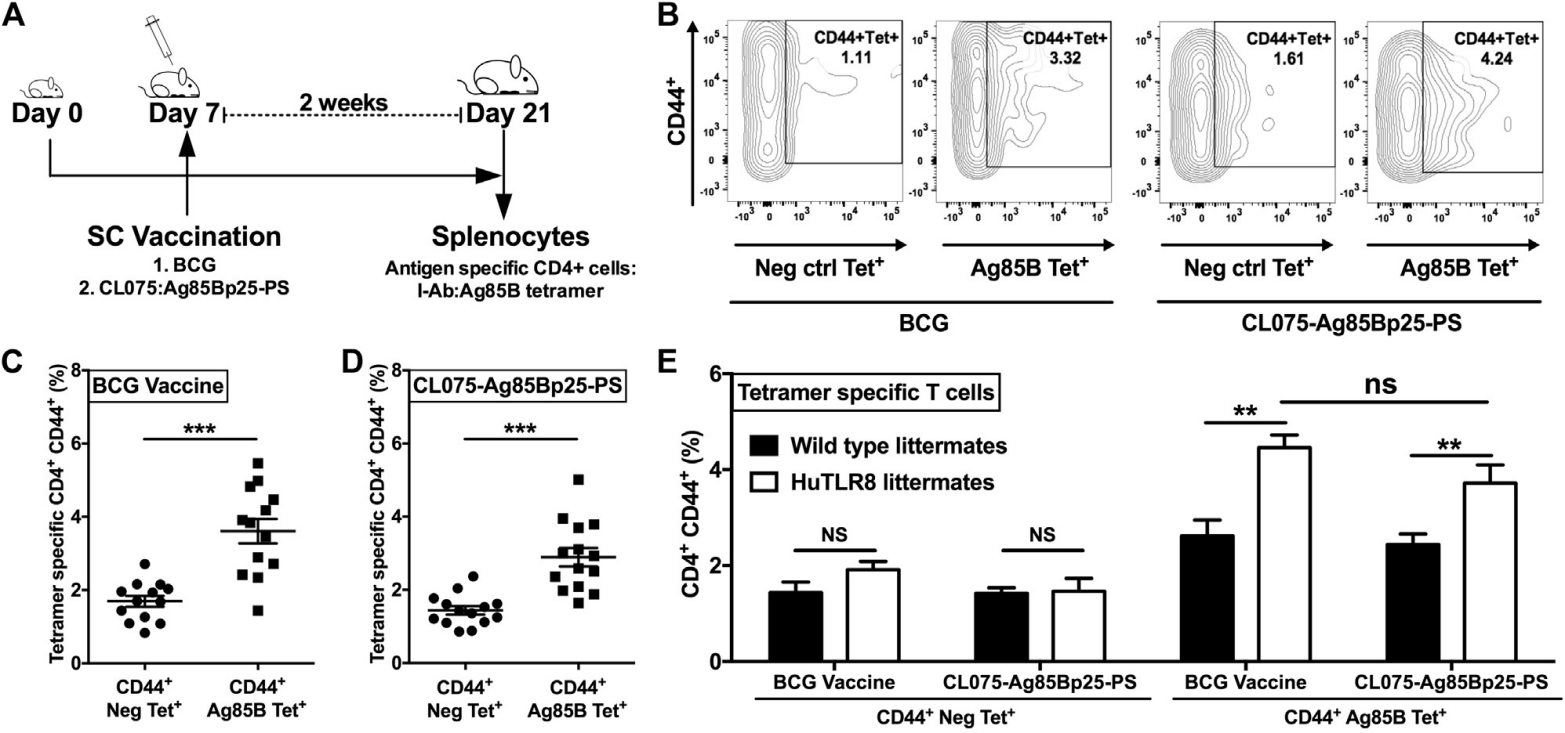
CL075:Ag85Bp25-PS and BCG induce comparable Ag85B-specific CD4^+^ effector T cells in the spleens of neonatal huTLR8Tg mice. **A,** Experimental design: 7-day-old huTLR8Tg and matched littermate WT mice were immunized subcutaneously and killed 14 days later before harvesting spleens and staining splenocytes with a control versus Ag85B_280–294_ MHCII tetramer and for surface marker expression. **B,** Representative flow cytometric plots showing Tet^+^ T cells as CD44^+^ Neg Tet^+^ or CD44^+^ Ag85B Tet^+^ cells gated on live CD4^+^ splenic lymphocytes of BCG- or CL075-Ag85Bp25-PS–vaccinated mice 2 weeks after immunization. **C** and **D,** Similar to BCG, CL075-Ag85Bp25-PS induced a significant increase in CD44^+^ Ag85B Tet^+^ cells compared with CD44^+^ Neg Tet^+^ cells. Results represent means ± SEMs (n = 14-15), with significance relative to unspecific control tetramer. **E,** Neonatal humanized TLR8 mice have greater numbers of splenic Ag85B-specific CD4^+^ T cells versus their WT littermates. The unpaired Mann-Whitney test was applied, and statistical significance was denoted as follows: **P < .01 and ***P < .001. *NS*, Not significant.

**Fig E13:**
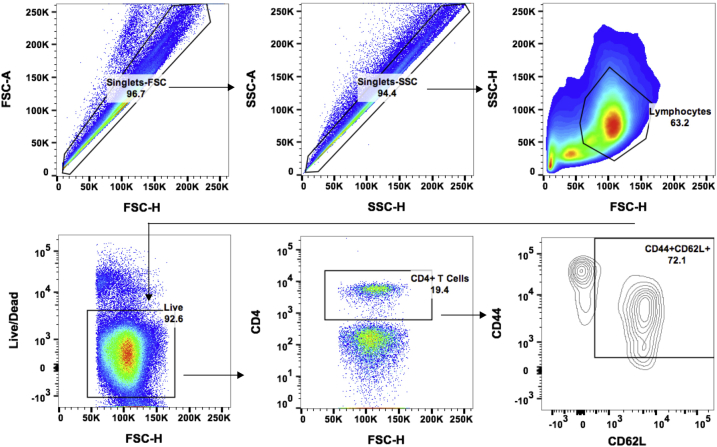


Next, we coloaded our CL075-PS formulation with the *M tuberculosis* Ag85Bp25 (CL075:Ag85Bp25-PS; Table I). Seven-day-old huTLR8Tg and matched littermate WT mice were vaccinated subcutaneously with either BCG or CL075:Ag85Bp25-PS and killed 14 days later (on day 21 of life; Fig 6, *A*). Spleens were collected, and the percentage of Tet^+^CD4^+^ T cells was determined by using a combination of expression of linage surface markers, staining for a negative control tetramer complexed with an unrelated antigen or a Ag85B_280–294_-complexed MHCII tetramer (see Fig E13 in this article's Online Repository at www.jacionline.org). We found that CL075-Ag85Bp25-PS induced robust induction of Ag85B-specific CD4^+^ effector T cells in the spleens of neonatal mice similar to BCG vaccination (Fig 6, *B*). CL075-Ag85Bp25-PS vaccination induced CD44^+^ Ag85B Tet^+^ cells in the spleen at significantly enhanced levels compared with CD44^+^ Neg Tet^+^ cells, which increased from approximately 1.69% to approximately 3.61% for BCG and from approximately 1.43% to approximately 3.00% for CL075-Ag85Bp25-PS, respectively (*P* < .001, with respect to the CD44^+^ Neg Tet^+^ groups; Fig 6, *C* and *D*). Importantly, no significant difference was noted between neonatal huTLR8Tg mice vaccinated with either BCG or CL075-Ag85Bp25-PS (*P* < .01; Fig 6, *E*), whereas significantly more Ag85B-specific CD4^+^ T cells were detected in the spleens of BCG or CL075-Ag85Bp25-PS-immunized huTLR8Tg mice versus WT littermates (*P* < .01; Fig 6, *E*).

## Discussion

Modern adjuvanted vaccine design integrates knowledge of formulation composition and insight into the mechanisms of immunostimulation to generate immune responses not otherwise achievable in relevant target populations.^11^, ^38^ New adjuvant formulations and antigen delivery systems might be key to tailoring vaccines to match the characteristics of a given vulnerable target population, such as newborns and infants.^2^ Here we have combined engineering and rational vaccine design approaches to develop a nanoparticle-based adjuvant and antigen delivery system designed to be active in human newborns and infants that mimics and in some instances exceeds the immunostimulatory effect of the live attenuated tuberculosis vaccine BCG on human neonatal DCs. We show that the assembled morphology of our block copolymers directed the *in vivo* uptake by APCs to allow targeting of diverse DC subsets. DCs are desirable targets because they are highly efficient at processing and inducing immunity against nanoscale pathogens and their components.^18^ Although previous studies have linked nanoparticle size, shape, and elasticity to enhanced circulation time and decreased clearance by the reticuloendothelial system,^39^, ^40^ to the best of our knowledge, our data are the first to demonstrate that chemically identical but morphologically different nanocarriers can be engineered for selective uptake by monocytic cell subsets, with key DC populations essential for vaccination preferring PEG_17_-*bl*-PPS_30_ PS.

Guided by our *in vivo* biodistribution results, we next developed DC-targeting PEG_17_-*bl*-PPS_30_ polymersomes loaded with CL075, a highly selective thiazoloquinoline TLR8 agonist. We have previously demonstrated that TLR8 agonists robustly activate human newborn leukocytes *in vitro*^13^, ^14^, ^41^ and that targeted delivery of TLR agonists to human and mouse DCs can amplify their adjuvanticity.^42^

Here, using formulation chemistry and without the need for extensive medicinal chemistry,^43^ we prepared polymersome formulations containing high concentrations (>5 mmol/L) of CL075 that were stable for several months. We demonstrated previously that IMQ-loaded polymersomes released their payloads after selective uptake by DCs and intracellular disruption for induced expression of cytokines and surface coreceptors.^21^ We noted similarly favorable cellular pharmacodynamic properties for CL075-PS, which in addition to stimulating adult DCs also induced T_H_1-polarizing responses from neonatal DCs. The vast majority of studies of newborn pattern recognition receptor agonist responses *in vitro* and *in vivo* describe impaired production of T_H_1 cytokines, such as IL-12p70, likely limiting the immunogenicity of such candidate adjuvants in vaccines targeting intracellular pathogens.^2^, ^44^ In this context our development of adjuvanted nanoparticle formulations with robust T_H_1-polarizing activity in early life is notable and might have translational implications Furthermore, our polymersome nanoparticle platform is versatile and amenable to codelivery of different antigens and adjuvants within the same formulation, as demonstrated by loading of either HIV-1-Gag protein or Ag85Bp25 with CL075.

By using *in vitro* human and murine DC assays, we benchmarked the immunomodulatory abilities of CL075-PS against several adjuvants and licensed pediatric vaccines. Although previous studies have investigated the *in vitro* stimulatory abilities of vaccines on human DCs,^45^ ours is the first to do so using both newborn and adult cells cultured in intact (eg, non–heat inactivated) autologous plasma, a rich source of age-specific immunomodulatory factors.^35^ Because injection site and systemic reactogenicity are key considerations in developing novel vaccines, *in vitro* human leukocyte studies might directly de-risk formulation selection for *in vivo* use.^46^, ^47^ We found that a novel TLR8 agonist nanoparticle formulation mimicked the BCG-induced adult-like maturation signature in neonatal DCs. CL075-PS induced a similar *in vitro* PGE_2_ production profile compared with BCG, a biomarker that might correlate with adjuvants/adjuvanted vaccine–associated reactogenicity *in vivo*.^36^ Of note, this adult-like immunomodulatory effect of CL075-PS on neonatal DCs corresponded with lower LDH release than BCG vaccine, suggesting reduced induction of cell death. CL075-PS not only matched the BCG-induced pattern for most innate DC responses measured but actually exceeded BCG with respect to inducing production of IL-12p70 and IFNγ, suggesting a strong capacity of this novel nanoparticle formulation to favor T_H_1 immunity in newborns. When coloaded with the *M tuberculosis* Ag85Bp25, CL075:Ag85Bp25-PS induced BCG-like antigen-specific CD4^+^ adaptive immune responses in humanized TLR8 transgenic neonatal mice *in vivo*.

Our studies suggest that nanoparticle polymersome formulations hold great promise in the development of new early-life vaccines targeting intracellular pathogens. For example, although BCG is largely safe and reduces the risk of disseminated early-life tuberculosis, disadvantages of this live attenuated vaccine include inconsistent production resulting in both type-by-type (eg, BCG-Denmark vs BCG-India) and lot-to-lot variability that can affect clinical efficacy,^48^ as well as the risk of disseminated BCG infection (“BCGosis”) in those with certain primary immunodeficiencies.^49^ Accordingly, novel vaccine formulations with a more practical production path that induce protection against disseminated tuberculosis in early life might be of substantial public health benefit. The ability of CL075-PS to activate neonatal MoDCs to levels similar to their adult counterparts is particularly noteworthy and suggests that this adjuvantation platform might have applicability for early-life immunization, including development of novel vaccines against intracellular pathogens, such as HIV, tuberculosis, and malaria.

In summary, by combining nanotechnology, age- and species-specific *in vitro* modeling, and benchmarking to licensed vaccines, our study provides a fresh paradigm for rational vaccine design to inform development of novel age-targeted neonatal and pediatric vaccines. We characterized the *in vitro* and *in vivo* immunostimulatory activities of a novel polymersome-based adjuvant formulation rationally designed for limited bioavailability and intracellular delivery of the thiazoquinolone TLR8 agonist CL075 to murine and human newborn and adult DCs. By benchmarking to licensed vaccines *in vitro*, we found that CL075-PS induced adult-like responses in neonatal DCs that matched or exceeded activation induced by BCG, an effective in neonatal vaccine. More broadly, this study outlines a rational vaccine design approach using controlled preparation of TLR agonist–polymer complexes of the desired properties, size, and antigen load, potentially limiting systemic toxicities through targeted delivery of vaccine complexes.

**Key messages**

• A rational vaccine design approach using age-specific *in vitro* and *in vivo* models produced highly defined small-molecule TLR8 agonist–encapsulating polymer complexes with properties, including size and antigen load, which are appropriate for inducing innate and adaptive immune responses.
• TLR8 agonist polymersome nanoparticles matched or exceeded the activating effects of the live attenuated BCG vaccine on human neonatal DCs *in vitro*, including T_H_ polarizing cytokine induction and upregulation of costimulatory molecules.
• When coloaded with the *M tuberculosis* Ag85Bp25, the TLR8 agonist–containing polymersome nanoparticles were comparable with BCG in inducing antigen-specific immune responses in human TLR8–expressing neonatal mice *in vivo*.

## Supplementary data

Video E1

Video still

Online Repository text

Table E1
