# Supplementary material for: Toll-like receptor 8 agonist nanoparticles mimic immunomodulating effects of the live BCG vaccine and enhance neonatal innate and adaptive immune responses
Source: J Allergy Clin Immunol. 2017 Nov;140(5):1339–50. doi: 10.1016/j.jaci.2016.12.985 (PMC5667586; doi:10.1016/j.jaci.2016.12.985)
Supplement: Video still [file mmc2.pdf]

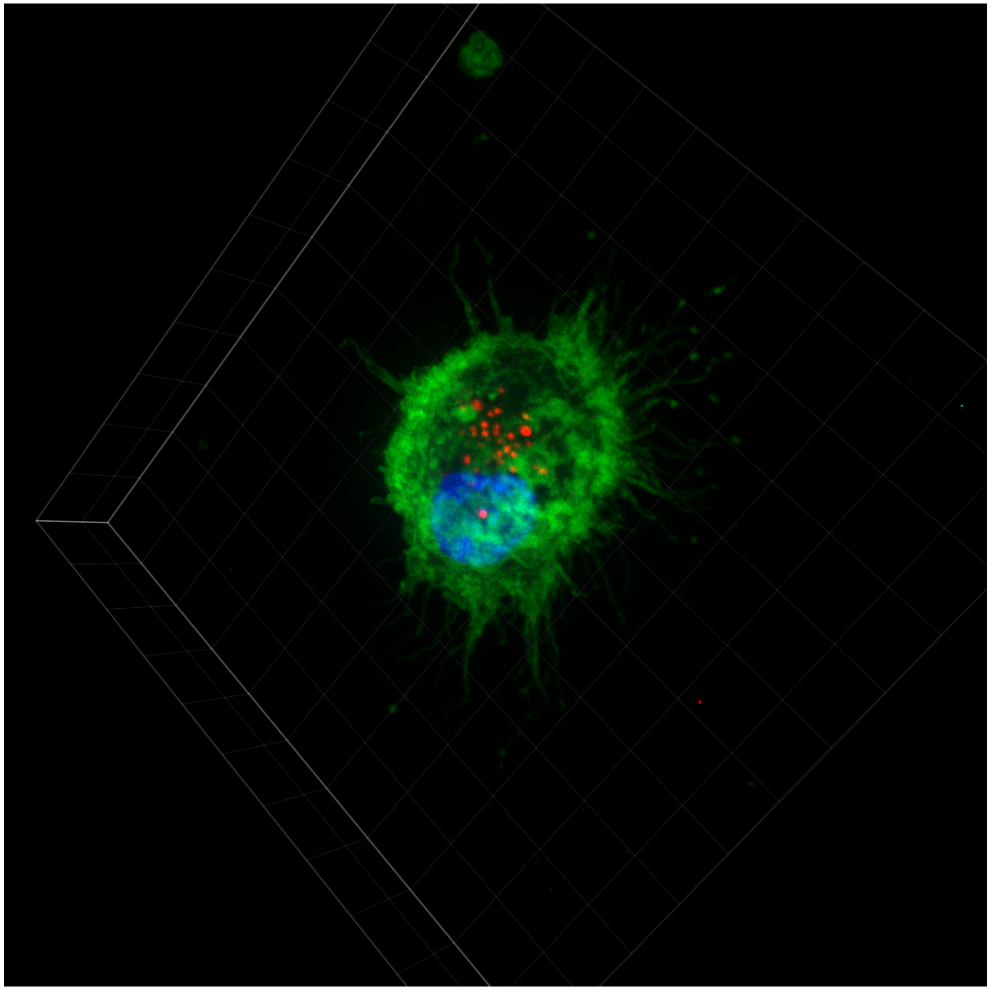

Video E1. Uptake of PS formulations by human DCs. Confocal microscopy of human adult DCs cultured with Bodipy-labeled PS at 68x oil immersion white light laser (405 for Dapi) for 4 hr. Cells were stained to identify the locations of PS (red), nucleus (blue) and HLA-DR (green).
