# Supplementary material for: Toll-like receptor 8 agonist nanoparticles mimic immunomodulating effects of the live BCG vaccine and enhance neonatal innate and adaptive immune responses
Source: J Allergy Clin Immunol. 2017 Nov;140(5):1339–50. doi: 10.1016/j.jaci.2016.12.985 (PMC5667586; doi:10.1016/j.jaci.2016.12.985)
Supplement: Online Repository text [file mmc3.docx]

**Repository text**

**Toll-like receptor 8 agonist nanoparticles mimic immunomodulating effects of the live BCG vaccine and enhance neonatal innate and adaptive immune responses**

David J. Dowling, PhD,^a,b^*, Evan A. Scott, PhD,^c,^*, Annette Scheid, MD,^a,b,d,e,^§ , Ilana Bergelson, BSc,^a^, Sweta Joshi, PhD,^a,e^, Carlo Pietrasanta, MD,^a,b,e,f^, Spencer Brightman, BA,^a,e^, Guzman Sanchez-Schmitz, MSc, PhD,^a,b,e^, Simon D. Van Haren, PhD,^a,b,e^, Jana Ninković, PhD,^a,b^, Dina Kats, BSc,^c^, Cristiana Guiducci, PhD,^g^, Alexandre de Titta, Ph,D,^h^, Daniel K. Bonner, PhD,^h^, Sachiko Hirosue, PhD,^h^, Melody A. Swartz, PhD,^h,i^, Jeffrey A. Hubbell, PhD,^h,i,^‡, Ofer Levy, MD, PhD,^a,b,e,2,^‡.

**Author affiliations:** ^a^Department of Medicine, Division of Infectious Diseases, Boston Children’s Hospital and ^b^Harvard Medical School, Boston, MA, 02115, USA, ^c^Department of Biomedical Engineering, Northwestern University, Evanston, IL, 60208, USA, ^d^Division of Newborn Medicine, Floating Hospital for Children, Tufts Medical Center, Boston, MA, 02111, USA, *^e^Precision Vaccine Program*, Divisions of a Infectious Diseases, Boston Children's Hospital, Boston, MA, 02115, USA, ^f^Neonatal Intensive Care Unit, Department of Clinical Sciences and Community Health, Fondazione IRCCS Ca' Granda Ospedale Maggiore Policlinico, University of Milan, Milan, Italy, ^g^Dynavax Technologies Corporation, Berkeley, CA, USA, ^h^Institute of Bioengineering, School of Life Sciences and School of Engineering, École Polytechnique Fédérale de Lausanne (EPFL), CH-1015 Lausanne, Switzerland, ^i^Institute for Molecular Engineering, University of Chicago, Chicago, IL 60637, USA.

Additional Footnotes:

*These authors contributed equally to this work.

‡These authors contributed equally to this work.

§Dr Scheid is currently affiliated with the Department of Pediatric Newborn Medicine, Brigham and Women’s Hospital, Boston, Mass.

**Corresponding authors:**

David J. Dowling, PhD,

Division of Infectious Diseases,

Enders Research Labs (Room 730),

Boston Children's Hospital,

300 Longwood Avenue, Boston, MA 02115

Phone: 617-919-2906

Email: david.dowling@childrens.harvard.edu

Evan A. Scott, PhD,

Department of Biomedical Engineering,

Northwestern University

Silverman Hall, 2170 Campus Drive, Room 4627,

Evanston IL, 60208

Phone: (847) 467-6719

Email: evan.scott@northwestern.edu

Ofer Levy MD, PhD,

Division of Infectious Diseases,

Enders Research Labs (Room 861.1),

Boston Children's Hospital,

300 Longwood Avenue, Boston, MA 02115

Office: 617-919-2904

Fax: 617-730-0255

E-mail: ofer.levy@childrens.harvard.edu

**Methods**

**Preparation of HIV-1 Gag p24 (HIV-Gag).** The preparation of HIV-1 Gag p24 (HIV-Gag) was based on previously published methods^1^. Briefly, to express HIV1 p24 in mammalian cells, codon optimized HIV1 p24 AY181195 (AIDS Research and Reference Reagent Program, Division of AIDS, National Institute of Allergy and Infectious Disease, NIH) modified to translate to the reference strain HXB2 (GenBank: K03455.1) was synthesized and cloned into pSecTagA (Invitrogen) using the HindIII and XbaI restriction enzyme sites. The protein was produced in serum-free suspension cultures of HEK293E cells by transient transfection in the presence of 3.75 mM valproic acid (Sigma-Aldrich) as described^2^. At Day 7 post-transfection, the cell culture medium was collected by centrifugation and concentrated using Amicon-Ultra Centrifugal Filter Units (10 kDa MWCO, Millipore) after pre-wetting the membrane with a solution of PBS pH 7.4 + 0.2 % Tween 20.  The protein was purified by size exclusion chromatography (HiLoad 16/60 75pg Superdex column on the ÄKTA Explorer system, GE Healthcare) in PBS pH 7.4. Protein identity was confirmed by SDS-PAGE and N-terminal sequence analysis. HIV-Gag p24 was stored at -80°C. Batch analyses by HPLC profiles showed that each batch contained a single peak in the macromolecular section of the chromatograms, consistent with a single protein composition of the formulations. The retention time of the peaks was closely similar for all batches (11.0 - 11.2 min) and consistent with the protein’s molecular weight (24 kDa). Three batches of HIV-Gag were well separated on the linear column employed and display no signs of non-specific interactions batches of macromolecular fraction of HIV-Gag.

**Synthesis and characterization of CL075-loaded polymersome nanoparticles.** Copolymers with hydrophilic block weight fractions of 0.28, 0.38, and 0.48 respectively assembled into PS, filomicelles, and spherical micelles (Fig 1, *A*), mimicking the diverse nanostructures of virions^3, 4^. Briefly, benzyl mercaptan was used to initiate the living polymerization of propylene sulfide. The terminal thiolate was end-capped with PEG-mesylate. Block copolymers were precipitated with cold methanol and the purity was verified by gel permeation chromatography (GPC) using Waters Styragel THF columns (HR 2, 3, and 4) with a tetrahydrofuran (THF) mobile phase via both refractive index and UV/vis detectors (Waters Corporation). All chemicals were purchased from Sigma-Aldrich. For studies requiring fluorescently tagged PEG-*bl*-PPS, co-polymers were synthesized by initiating the polymerization of propylene sulfide polymerization with PEG-thiol and end-capping the PPS with bodipy 577/618 maleimide (Invitrogen). Polymersomes were formed and loaded with CL075 (2-propylthiazolo[4,5-c]quinolin-4-amine; formula, C13H13N3S; m.w., 243, also called 3M-002^5^) using thin film rehydration^3^. PEG_17_-bl-PPS_30_ was dissolved in dichloromethane and desiccated for 2 hr within piranha-etched glass vials. The resulting thin films were rehydrated by overnight rotation at 4°C in 50 mM PBS containing either CL075, HIV-1-Gag protein, or the mycobacteria Ag85B peptide 25, Sequence (N to C): FQDAYNAAGGHNAVF (15 aa) (Ag85Bp25) (Biomatik, Wilmington, Delaware, USA). The multilamellar polymersomes were extruded through 0.2 μm and then 0.1 μm nucleopore track-etched membranes (Whatman) to obtain nanoparticles with diameters less than 200 nm. Size exclusion chromatography with Sepharose CL-6B resin and a PBS mobile phase was used to verify that there was no free CL075 present after purification with Sephadex LH-20 resin. Both free and encapsulated IMQ were quantified by degrading the polymersomes via oxidation with 0.5% solutions of hydrogen peroxide and quantifying the amount of free molecules of the CL075 using UV/fluorescence HPLC (Waters Alliance 2695, Waters Corporation). The amount of HIV-Gag loaded within polymersomes was determined by disrupting vesicles with 0.5% (v/v) solutions of Triton-X 100 and quantifying the amount of free protein in the supernatant with ELISA.

**Particle size determination and endotoxin testing.** After preparation, polymersome particle size was determined by dynamic light scattering (DLS), with suspensions of PS in PBS (0.1 mg/ml) analyzed with a Zetasizer Nanoseries instrument (Malvern Nano-ZS, l 1⁄4 532 nm; Westborough, MA). Particle size data indicate scattering intensity distributions (z-average). Stability of PS at 4°C was also characterized over a 6 month period. At the time of production, HIV-Gag p24 and polymersomes endotoxin levels were tested using HEK-Blue TLR4 (InvivoGen, San Diego, CA). Before use, PS, HIV-Gag, Gag2 control peptide and Alum were tested for endotoxin levels by *Limulus* amoebocyte lysate (LAL) assay according to the manufacturer’s instructions (Charles River; Boston, MA). All reagents used for tissue culture tested < 1 EU/ml.

**Human blood.** Peripheral blood was collected from healthy adult volunteers, while human newborn cord blood was collected immediately after Cesarean section delivery of the placenta. Births to HIV-positive mothers were excluded. Human experimentation guidelines of the U.S. Department of Health and Human Services, The Brigham & Women’s Hospital, and Boston Children’s Hospital were observed, following protocols approved by the local institutional review boards. Human blood was anti-coagulated with 20 units/ml pyrogen-free sodium heparin (American Pharmaceutical Partners, Inc., Schaumberg, IL). Blood products were kept at room temperature and processed within 4 hr of collection.

**Flow cytometry and confocal microscopy for human DCs.** Following stimulation, MoDCs were re-suspended in staining buffer (1 x PBS, 0.5% [v/v] human serum albumin) and stained for 30 min at 4°C in the dark (1 x 10^5^ cells/per staining) with fluorophore-labeled antibodies (CD83/Allophycocyanin/Clone HB15e, CD86/phycoerythrin/Clone 2331, CD40/FITC/ 5C3, CCR7/V450/Clone 150503 and HLA-DR/PerCP-Cy5.5/Clone L243 (BD Biosciences)). Cells were then centrifuged (500 x g, 10 min), washed and fixed (1% [v/v] paraformaldehyde) prior to flow cytometry acquisition using a LSRII flow cytometer employing BD FACSDiva software (BD Biosciences) and data analyzed using FlowJo software (Tree Star, Inc., Ashland, OR). Compensation settings were assessed using BD CompBead particles (BD Biosciences) and compensation calculated and applied in FACSDiva software. > 5,000 events per sample were acquired. For Confocal microscopy, newborn and adult MoDCs were treated for the indicated times with fluorescently labeled PS (Bodipy). Cells were fixed, labeled with fluorophore-labeled antibodies for DAPI, HLA-DR, actin, early and late endosome markers EEA and LAMP-1, and imaged via fluorescent microscopy (Axio Vert, Zeiss Germany).

**Generation of neonatal bone marrow derived dendritic cells (BMDCs)**

Mice were maintained under specific pathogen-free conditions. BMDCs were generated from newborn (7 days old) and adult (8 - 12 weeks old) C57BL/6 mice with an adaptation of previously published methods^6^. Briefly, mice were sacrificed and legs removed, cutting away the entire leg above the hip. Into a sterile petri dish under a BSL-2 cabinet, bones were surgically cleaned from surrounding tissue using small forceps and curved, blunt scissors, extremities of tibiae and femurs were trimmed with sterile scissors and bone marrow flushed into a 50 ml collection tube through a 70 μm nylon mesh strainer (Fisher Scientific) using a sterile 20 ml syringe filled with RPMI and equipped with a 27G needle. Cells were centrifuged at 500 x g for 10 minutes and resuspended in fresh RPMI.  Cells were plated into non tissue culture – treated 100 mm Petri dishes (Falcon) at a concentration of 3 x 10^5^ cells/ml in 10 ml/plate of complete culture medium (RPMI 1640 plus 10% heat inactivated fetal bovine serum (HyClone - Thermo Scientific), 50 μM 2-Mercaptoethanol (Gibco - Thermo Scientific), 2 mM L-glutamine, 100 U/ml penicillin/streptomycin (Thermo Scientific) and 20 ng/ml of recombinant murine granulocyte-macrophage colony-stimulating factor (rmGM-CSF) (R&D systems). Plates were incubated in humidified atmosphere at 37˚C, 5% CO_2_ for a total of 6-7 days, with one supplement of 10 ml of complete culture medium added on day 3. On day 6-7, loosely adherent cells were harvested, by washing the plate extremely gently with RPMI. Adherent cells were discarded. The purity of immature BMDCs from adult and newborn mice were investigated by flow cytometry with the following monoclonal antibodies: BV421-conjugated anti-CD11c (clone N418), APC-conjugated anti-I-A[b] (clone M5/114 (both from Biolegend), with the fixable viability dye eFluor 780 (eBioscience) used to exclude dead cells. For stimulation experiments, immature BMDCs generated from 7 days old mice were used. BMDCs were plated in round bottom 96-wells non tissue culture-treated plates at the density of 10^5^ cells/well in 200 μl of fresh complete culture medium, with the appropriate stimulus at the concentrations indicated in each figure legend. Plates were incubated for 24 hours, centrifuged at 500 x g for 10 minutes and ~160 μl/well of supernatant were carefully aspirated with a multichannel pipette without disturbing the cell pellet. Supernatants were assayed by ELISA for TNF, IL-6 and IL-1β with commercially available kits (R&D Systems, Minneapolis, MN), according to the manufacturer instructions.

**Repository Figure Legends**

**Fig E1. Polymersomes preferentially associate with splenocytes and myeloid cells.** Amine functionalized PEG_17_-*bl*-PPS_30_ block copolymers were conjugated to fluorescent Dy647-N-hydroxysuccinimide and assembled in the presence of block copolymers engineered to form PS vesicles (PS; PEG_17_-*bl*-PPS_30_), filomicelles (F; PEG_45_-*bl*-PPS_44_) or spherical micelles (M; PEG_44_-*bl*-PPS_29_). Murine popliteal lymph nodes and spleen were harvested and processed into single cell suspensions. (**A**) Gating strategy for pDCs, monocytes and granulocytes in the spleen and LN. (**B**) Gating strategy for DC compartment in the spleen and LN. Cell populations defined in the manuscript were gated as positive for polymer constructs-Dy647 (in light blue) or negative (in black). Fluorescently-labeled PSs (Dy647) were subsequently incubated with lymph node (**C**) or spleen (**D**) suspensions for 24h. (**E**) Percentages of key phagocyte populations that associated with PEG-*bl*-PPS filaments (F), vesicular polymersomes (PS), and micelles (M). Flow cytometric analysis was completed post incubating (mean ± SEM, n = 5 - 8).

**Fig E2. Schematic representation of the creation of TLR8 agonist encapsulating polymersome nanoparticles.** Step 1: Block copolymers composed of poly(ethylene glycol) and poly(propylene sulfide) self-assemble into oxidation-sensitive polymersomes in aqueous solutions. Step 2: Block copolymers are precipitated with cold methanol and the Step 3: purity was verified by gel permeation chromatography (GPC). Step 4: Polymersomes are formed and loaded with molecules using thin film rehydration. Step 5: Polymersomes with low polydispersity were obtained by extrusion through nanoporous membranes. Step 6: Purification from unloaded molecules employed size exclusion chromatography using Sepharose CL-6B resin, and Step 7: verified with UV/fluorescence HPLC. Step 8: Average size, polydispersity, endotoxin content and stability was determined at time of production and again at time of use. Polymersome size distributions were obtained with dynamic light scattering (DLS). DLS analysis was performed with polymersome suspensions in PBS (0.1 mg/ml).

**Fig E3. Intrinsic fluorescence of CL075 enables monitoring encapsulation concentrations after release from polymersomes.** Representative data of method used to quantify CL075 concentrations using fluorescence HPLC (with Sepharose 6B resin). (**A**) Polymersomes elute from the size exclusion column between 5 and 8 min while free CL075 eluted between 14 and 16 min. (**B**) Detection of TLR agonist release from polymersomes after 48 h incubation with 0.5% hydrogen peroxide is benchmarked against known control concentrations of CL075.

**Fig E4. Diameter of CL075-loaded polymersome nanoparticles over six months of storage at 4°C.** Dynamic light-scattering measurement of PS (PEG_17_-*bl*-PPS_30_) analyzed with the ZetaSizer Nano at time of production (day 0) and following storage at 4°C in PBS for ~180 days. Data represent mean ± SEM for replicate measurements (n = 3). Unpaired Students t-test was applied at each time and statistical significances are denoted as follows: ** p < 0.01. NS, not significant.

**Fig E5. PS nanoparticles are internalized by human DCs.** (**A**) Confocal microscopy of human newborn and adult DCs cultured with Bodipy labeled PS nanoparticles at 68 x oil immersion white light laser (405 for Dapi) at 1, 2 and 4 hr time points. Cells were stained to identify the locations of PS (red), nucleus (blue) and HLA-DR (green). (**B**) PS uptake and trafficking within DCs. Confocal microscopy at 68 x oil immersion white light laser (405 for Dapi) at 30 minutes, 4 hr and 24 hr time points. Cells were stained to identify the locations of PS (red), actin (purple), nucleus (blue) and EEA or Lamp-1 (green).

**Fig E6. CL075-PSs induce adult-level TNF production from human newborn DCs.** Newborn and adult MoDCs were cultured in 10% autologous plasma (vol/vol) and stimulated for 24 hr with PSs, free CL075, CL075-PSs, BCG or Alum-adjuvanted vaccines prior to collection of supernatants for TNF ELISA (mean ± SEM, n = 8 - 14). For analyses at individual treatments (e.g. RPMI control vs. free CL075), unpaired Mann-Whitney test was applied at each concentration and statistical significances are denoted as follows: * p < 0.05, ** p < 0.01 and *** p < 0.001. For comparisons between overall groups (i.e., newborn vs. adult), significance denoted as +p < 0.05. NS, not significant.

**Fig E7. CL075-PS induces concentration-dependent cytokine production from human newborn DCs.** (**A**) Radar plots of pg/ml cytokine concentrations induced by PS, CL075, CL075-PS (0.1, 1, 5, 10 µM, increasing shades of blue), compared to control (RPMI, black). (**B**) Radar plots of immune polarizing cytokine production from pulsed human newborn DCs, represented as fold-change over RPMI unstimulated control. Both data sets represent cytokine production after 24 hr culture (mean ± SEM, n = 6).

**Fig E8. CL075-PS induces concentration-dependent cytokine production from human adult DCs.** (**A**) Radar plots of pg/ml cytokine concentrations induced by PS, CL075, CL075-PS (0.1, 1, 5, 10 µM, increasing shades of red), compared to control (RPMI, black). (**B**) Radar plots of immune polarizing cytokine production from pulsed human adult DCs, represented as fold-change over RPMI unstimulated control. Both data sets are after 24 hr culture (mean ± SEM, n = 6).

**Fig E9. Free CL075, CL075-PS and CL075:Gag-PS induce closely similar cytokine production from human newborn and adult DCs.** (**A**) newborn and (**B**) adult MoDC responses to increasing concentrations of CL075 (black), CL075-PS (green) or CL075:Gag-PS (red) (0.1, 1, 5, 10 µM). Radar plots of cytokine production are represented as fold-change over the RPMI unstimulated control after 24 hr culture (mean ± SEM, n = 6).

**Fig E10. TLR8 agonist encapsulating polymersomes induce greater IL-12p70 production from human DCs than live BCG vaccine or LPS.** Newborn (**A**) and adult (**B**) MoDCs were cultured in 10% autologous plasma (vol/vol) and stimulated for 24 hr with free CL075 (blank), CL075-PS (green), CL075:Gag-PS (red) (each with a CL075 concentration of 1, 5, 10 µM), or BCG (blue) (each at 1:1000, 1:100, 1:10 vol/vol). IL-12p70 production was measured by multiplex assay. (**C**) CL075-PSs (0.1, 1, 5, 10 µM) induce concentration-dependent IL-12p70 responses in newborn DCs matching those in adult DCs. Comparison is made to LPS (100 ng/ml). For analyses at individual treatments (e.g. RPMI control vs. 0.1 µM CL075), unpaired Mann-Whitney test was applied at each concentration and statistical significance denoted as follows: * p < 0.05, ** p < 0.01 and *** p < 0.001. For comparisons between overall groups (i.e., newborn vs. adult), significance denoted as + p < 0.05. NS, not significant. Graphs depict mean ± SEM, n = 5 - 6.

**Fig E11. BMDCs from neonatal humanized TLR8 mice have a more robust and specific innate immune response to TLR8 stimulation than their WT littermates.** (**A**) Relative huTLR8TgCL8 gene expression from 5 separate WT, and 5 huTLR8 matched littermate 7 day old mice, derived from 3 separate litters. (**B-D**) The highly specific TLR8 agonist TL8-506 were used to demonstrate the TLR8-specific cytokine responses of huTLR8 mice as compared to their WT littermates. (E-G) Conversely, DCs from both huTLR8 and WT pups respond equally to the highly specific TLR7 agonist CL264. Cytokine data sets are after 24 hr culture (mean ± SEM, *n* = 5). For comparisons between groups (i.e., WT vs. huTLR8), significance denoted as follows: ** p < 0.01 and *** p < 0.001.

**Fig E12. Similar to BCG, CL075-PS induces robust activation of BMDCs from neonatal humanized TLR8 mice**. (**A**) BMDCs from 7 day old huTLR8 mice were stimulated for 24 hr with fixed concentrations of MPLA or LPS (both 100 ng/ml) or increasing concentrations of free CL075 (dashed blue line) or CL075-PS (green). (**B-D**) BMDCs stimulated for 24 hr as above or with fixed concentrations of Alum (5, 50, 500 µg/ml), BCG, PCV13 or HBV (each at 1:1000, 1:100, 1:10 vol/vol) (mean ± SEM, n = 8).

**Fig E13. Representative flow cytometry plots outlining the gating strategy used to identify CD4+ T cells in mouse spleens post vaccination.** CD44^+^ tetramer^+^ cells were identified using a gating strategy based on singlets, FSC×SSC lymphocyte characteristics, live cells and CD4 expression. Here, PE-conjugated CD62L antibody is shown in place of the Tet^+^ PE stain, with T central memory identified as CD62L^hi^, CD44^hi^ cells. About 5 x 10^6^ cells were stored for each sample acquired on LSR II flow cytometer (BD biosciences); data analysis was performed using FlowJo software (TreeStar, OR, USA).

**References**

E1. Gamble TR, Yoo S, Vajdos FF, von Schwedler UK, Worthylake DK, Wang H, et al. Structure of the carboxyl-terminal dimerization domain of the HIV-1 capsid protein. Science 1997; 278:849-53.

E2. Baldi L, Hacker DL, Meerschman C, Wurm FM. Large-scale transfection of mammalian cells. Methods Mol Biol 2012; 801:13-26.

E3. Scott EA, Stano A, Gillard M, Maio-Liu AC, Swartz MA, Hubbell JA. Dendritic cell activation and T cell priming with adjuvant- and antigen-loaded oxidation-sensitive polymersomes. Biomaterials 2012; 33:6211-9.

E4. Cerritelli S, O'Neil CP, Velluto D, Fontana A, Adrian M, Dubochet J, et al. Aggregation behavior of poly(ethylene glycol-bl-propylene sulfide) di- and triblock copolymers in aqueous solution. Langmuir 2009; 25:11328-35.

E5. Gorden KB, Gorski KS, Gibson SJ, Kedl RM, Kieper WC, Qiu X, et al. Synthetic TLR agonists reveal functional differences between human TLR7 and TLR8. J Immunol 2005; 174:1259-68.

E6. Lutz MB, Kukutsch N, Ogilvie AL, Rossner S, Koch F, Romani N, et al. An advanced culture method for generating large quantities of highly pure dendritic cells from mouse bone marrow. J Immunol Methods 1999; 223:77-92.
