# Supplementary material for: Toll-like receptor 8 agonist nanoparticles mimic immunomodulating effects of the live BCG vaccine and enhance neonatal innate and adaptive immune responses
Source: J Allergy Clin Immunol. 2017 Nov;140(5):1339–50. doi: 10.1016/j.jaci.2016.12.985 (PMC5667586; doi:10.1016/j.jaci.2016.12.985)
Supplement: Table E1 [file mmc4.docx]

| **Table E1. CL075-PS induce robust up-regulation of MoDC co-stimulatory molecules. Surface expression of co-stimulatory molecules and HLA was determined by flow cytometry and analyzed as fold-change in mean fluorescent intensity (MFI) vs. vehicle control (mean ± SEM, n = 5 - 8).** | | | | | | | | | | | | | | | | | | | | |
| --- | --- | --- | --- | --- | --- | --- | --- | --- | --- | --- | --- | --- | --- | --- | --- | --- | --- | --- | --- | --- |
| **Measurement** | **Newborn** | | | | | | | | | | **Adult** | | | | | | | | | |
|  | **CD40** | | **CD80** | | **CD86** | | **CCR7** | | **MHCII** | | **CD40** | | **CD80** | | **CD86** | | **CCR7** | | **MHCII** | |
|  | **Mean** | **SD** | **Mean** | **SD** | **Mean** | **SD** | **Mean** | **SD** | **Mean** | **SD** | **Mean** | **SD** | **Mean** | **SD** | **Mean** | **SD** | **Mean** | **SD** | **Mean** | **SD** |
| RPMI | 1.00 | 0.00 | 1.00 | 0.00 | 1.00 | 0.00 | 1.00 | 0.00 | 1.00 | 0.00 | 1.00 | 0.00 | 1.00 | 0.00 | 1.00 | 0.00 | 1.00 | 0.00 | 1.00 | 0.00 |
| MPLA (100ng/ml) | 1.69 | 0.54 | 2.09 | 0.20 | 1.73 | 0.40 | 1.41 | 0.22 | 1.69 | 1.05 | 1.68 | 0.77 | 2.57 | 0.77 | 1.65 | 0.58 | 1.32 | 0.08 | 1.28 | 0.54 |
| PS (empty) | 1.06 | 0.24 | 1.08 | 0.13 | 1.31 | 0.59 | 1.05 | 0.07 | 1.15 | 0.51 | 1.04 | 0.13 | 1.05 | 0.10 | 1.12 | 0.22 | 1.05 | 0.11 | 1.03 | 0.16 |
| CL075 (0.1 μM) | 1.13 | 0.38 | 1.14 | 0.33 | 1.05 | 0.16 | 1.04 | 0.07 | 1.21 | 0.57 | 1.00 | 0.07 | 1.02 | 0.08 | 1.06 | 0.08 | 1.01 | 0.09 | 1.04 | 0.10 |
| CL075 (1 μM) | 1.88 | 0.62 | 3.00 | 1.65 | 1.89 | 0.86 | 1.20 | 0.17 | 1.47 | 0.61 | 1.79 | 0.73 | 2.89 | 2.12 | 1.57 | 0.41 | 1.28 | 0.24 | 1.19 | 0.15 |
| CL075 (5 μM) | 2.79 | 1.31 | 4.23 | 1.98 | 2.20 | 0.83 | 2.83 | 1.95 | 1.61 | 0.97 | 3.28 | 1.45 | 4.29 | 3.23 | 2.18 | 0.18 | 1.85 | 0.38 | 1.53 | 0.27 |
| CL075 (10 μM) | 3.33 | 1.27 | 5.33 | 1.62 | 2.46 | 0.62 | 2.70 | 1.82 | 1.65 | 1.06 | 3.67 | 1.15 | 4.89 | 2.52 | 2.23 | 0.41 | 2.25 | 0.30 | 1.59 | 0.15 |
| PS-CL075 (0.1 μM) | 1.19 | 0.17 | 1.18 | 0.16 | 1.27 | 0.30 | 1.12 | 0.09 | 1.15 | 0.37 | 1.24 | 0.36 | 1.37 | 0.73 | 1.25 | 0.19 | 1.04 | 0.14 | 1.10 | 0.17 |
| PS-CL075 (1 μM) | 1.63 | 0.52 | 2.61 | 1.26 | 1.67 | 0.62 | 1.23 | 0.09 | 1.22 | 0.42 | 1.65 | 0.63 | 2.65 | 1.71 | 1.58 | 0.42 | 1.25 | 0.13 | 1.14 | 0.20 |
| PS-CL075 (5 μM) | 2.60 | 0.54 | 4.47 | 1.71 | 2.32 | 0.86 | 2.25 | 0.91 | 1.41 | 0.51 | 2.89 | 1.58 | 4.58 | 3.81 | 2.22 | 0.51 | 2.04 | 0.44 | 1.42 | 0.26 |
| PS-CL075 (10 μM) | 2.78 | 0.64 | 4.75 | 1.38 | 2.38 | 0.72 | 2.68 | 1.70 | 1.36 | 0.57 | 3.18 | 0.81 | 4.32 | 1.78 | 2.26 | 0.32 | 2.27 | 0.77 | 1.45 | 0.18 |
| BCG (1:1000) | 1.12 | 0.51 | 1.67 | 0.57 | 1.35 | 0.51 | 1.58 | 0.26 | 0.95 | 0.38 | 1.17 | 0.33 | 1.46 | 0.15 | 1.41 | 0.66 | 1.65 | 0.51 | 1.18 | 0.65 |
| BCG (1:100) | 1.63 | 0.33 | 2.41 | 0.95 | 1.91 | 0.28 | 2.01 | 0.42 | 1.58 | 1.14 | 1.20 | 0.23 | 2.17 | 0.26 | 1.48 | 0.58 | 2.03 | 0.54 | 1.05 | 0.53 |
| BCG (1:10) | 1.47 | 0.43 | 2.50 | 2.09 | 2.09 | 0.71 | 3.51 | 0.75 | 1.40 | 0.73 | 1.40 | 0.19 | 3.07 | 1.28 | 1.92 | 0.83 | 4.35 | 0.69 | 1.09 | 0.55 |
| Alum (5 μg/ml) | 0.64 | 0.15 | 0.76 | 0.21 | 1.25 | 0.45 | 4.08 | 3.23 | 1.03 | 0.63 | 0.87 | 0.39 | 1.08 | 0.12 | 1.26 | 0.23 | 2.31 | 0.34 | 1.01 | 0.34 |
| Alum (50 μg/ml) | 0.46 | 0.23 | 0.66 | 0.20 | 1.28 | 0.52 | 2.08 | 0.90 | 0.91 | 0.60 | 0.69 | 0.22 | 0.77 | 0.09 | 1.25 | 0.71 | 2.30 | 1.44 | 0.85 | 0.36 |
| Alum (500 μg/ml) | 0.28 | 0.21 | 0.41 | 0.25 | 0.74 | 0.78 | 1.45 | 0.57 | 0.21 | 0.15 | 0.37 | 0.22 | 0.41 | 0.17 | 0.42 | 0.18 | 1.53 | 0.45 | 0.27 | 0.14 |
| PCV13 (1:1000) | 1.02 | 0.35 | 0.89 | 0.18 | 1.60 | 0.35 | 1.18 | 0.65 | 1.42 | 0.79 | 1.04 | 0.16 | 1.07 | 0.10 | 1.60 | 0.56 | 1.08 | 0.24 | 1.17 | 0.20 |
| PCV13 (1:100) | 0.50 | 0.26 | 0.52 | 0.28 | 1.05 | 0.56 | 2.91 | 1.29 | 1.09 | 0.83 | 0.76 | 0.30 | 0.85 | 0.18 | 1.73 | 1.17 | 2.47 | 1.24 | 1.17 | 0.35 |
| PCV13 (1:10) | 0.32 | 0.23 | 0.44 | 0.26 | 1.04 | 0.54 | 2.30 | 0.66 | 0.74 | 0.60 | 0.57 | 0.19 | 0.66 | 0.05 | 1.31 | 1.18 | 2.65 | 1.42 | 0.61 | 0.13 |
| HBV (1:1000) | 1.03 | 0.36 | 1.02 | 0.18 | 1.23 | 0.28 | 3.06 | 3.32 | 1.56 | 1.20 | 1.01 | 0.15 | 0.99 | 0.07 | 1.42 | 0.56 | 1.82 | 0.93 | 1.17 | 0.33 |
| HBV (1:100) | 0.84 | 0.25 | 0.95 | 0.22 | 1.90 | 0.59 | 1.85 | 0.97 | 1.69 | 0.88 | 1.07 | 0.29 | 1.08 | 0.21 | 1.79 | 0.79 | 2.28 | 1.07 | 1.38 | 0.25 |
| HBV (1:10) | 0.47 | 0.23 | 0.54 | 0.15 | 1.48 | 0.75 | 1.72 | 1.00 | 1.02 | 0.67 | 0.69 | 0.13 | 0.68 | 0.09 | 1.13 | 0.45 | 2.13 | 1.32 | 1.03 | 0.75 |
